# Exosomes from metastatic cancer cells transfer amoeboid phenotype to non-metastatic cells and increase endothelial permeability: their emerging role in tumor heterogeneity

**DOI:** 10.1038/s41598-017-05002-y

**Published:** 2017-07-05

**Authors:** Odessa Schillaci, Simona Fontana, Francesca Monteleone, Simona Taverna, Maria Antonietta Di Bella, Dolores Di Vizio, Riccardo Alessandro

**Affiliations:** 1 Department of Biopathology and Medical Biotechnologies, University of Palermo, Palermo, Italy; 2 Institute of Biomedicine and Molecular Immunology (IBIM), National Research Council, Palermo, Italy; 3 Division of Cancer Biology and Therapeutics, Departments of Surgery, Biomedical Sciences and Pathology and Laboratory Medicine, Samuel Oschin Comprehensive Cancer Institute, Los Angeles, CA USA

## Abstract

The goal of this study was to understand if exosomes derived from high-metastatic cells may influence the behavior of less aggressive cancer cells and the properties of the endothelium. We found that metastatic colon cancer cells are able to transfer their amoeboid phenotype to isogenic primary cancer cells through exosomes, and that this morphological transition is associated with the acquisition of a more aggressive behavior. Moreover, exosomes from the metastatic line (SW620Exos) exhibited higher ability to cause endothelial hyperpermeability than exosomes from the non metastatic line (SW480Exos). SWATH-based quantitative proteomic analysis highlighted that SW620Exos are significantly enriched in cytoskeletal-associated proteins including proteins activating the RhoA/ROCK pathway, known to induce amoeboid properties and destabilization of endothelial junctions. In particular, thrombin was identified as a key mediator of the effects induced by SW620Exos in target cells, in which we also found a significant increase of RhoA activity. Overall, our results demonstrate that in a heterogeneous context exosomes released by aggressive sub-clones can contribute to accelerate tumor progression by spreading malignant properties that affect both the tumor cell plasticity and the endothelial cell behavior.

## Introduction

Human tumors display a significant intratumor heterogeneity that influences their metastatic potential and therapeutic resistance. Tumor heterogeneity is mainly the result of genetic instability. However, the behavior of individual tumor cells can be further increased by epigenetic alterations, which are key factors in the formation of the tumor initiating cancer cell subpopulations^1, 2^. Intravital microscopy techniques, in a cancer living mouse model, have shown that the existence of few individual cells with aggressive molecular features within a tumor is sufficient to support cancer progression^3^. Over recent years, a growing number of studies suggest that the tumor microenvironment (TME), which contributes to a functional crosstalk between different cell types, plays an important role in determining the heterogeneity observed within and across tumors^4^. This has resulted in an increased understanding of the crosstalk that occurs between malignant cells and their microenvironment^5–10^. However, a number of major questions remain unanswered, underscoring the need to better characterize the steps of tumor progression, and thereby to identify new and effective ways of treating metastatic disease. Our group and others have demonstrated that cancer cells release oncogenic cargo in exosomes, which play a crucial role in the crosstalk between cells and TME^11–14^. Exosomes are nanometer-sized vesicles (40–100 nm diameter) of endocytic origin that are released by different cell types under both normal and pathological conditions. They function as cell free messengers that could potentially affect tumor heterogeneity^15^, due to the nature of the molecules (proteins, mRNAs, miRNAs and lipids) that they transport. Tumor cells actively shed exosomes into their surrounding microenvironment and these vesicles have pleiotropic functions in the regulation of tumor growth and progression, immune escape, tumor invasion, neovascularization, and metastasis^16^. In addition to effects exerted within the primary TME, tumor-derived exosomes (TDEs) play a crucial role in the establishment of the pre-metastatic niche^16^ by preparing lymph-node and new secondary sites for metastases^14^. TDEs can stimulate the secretion of growth factors, cytokines and angiopoietic factors by stroma cells, induce the proliferation of endothelial cells, thus promoting angiogenesis and metastasis in other organs^12, 17^. However, if and how TDEs can affect cell plasticity in the heterogeneous context of the primary tumor, thus spreading aggressive phenotype to less aggressive tumor cells and functionally affecting other components of the TME has not been elucidated yet.

Here, we demonstrate that exosomes derived from cells with high metastatic potential can modulate phenotypic plasticity in less aggressive cancer cells and elicit structural alterations of endothelial cells in a RhoA/ROCK dependent fashion. This ultimately contributes to create a permissive microenvironment for tumor dissemination.

## Results

### Characterization of SW480 and SW620-cell derived exosomes

**Figure 1:**
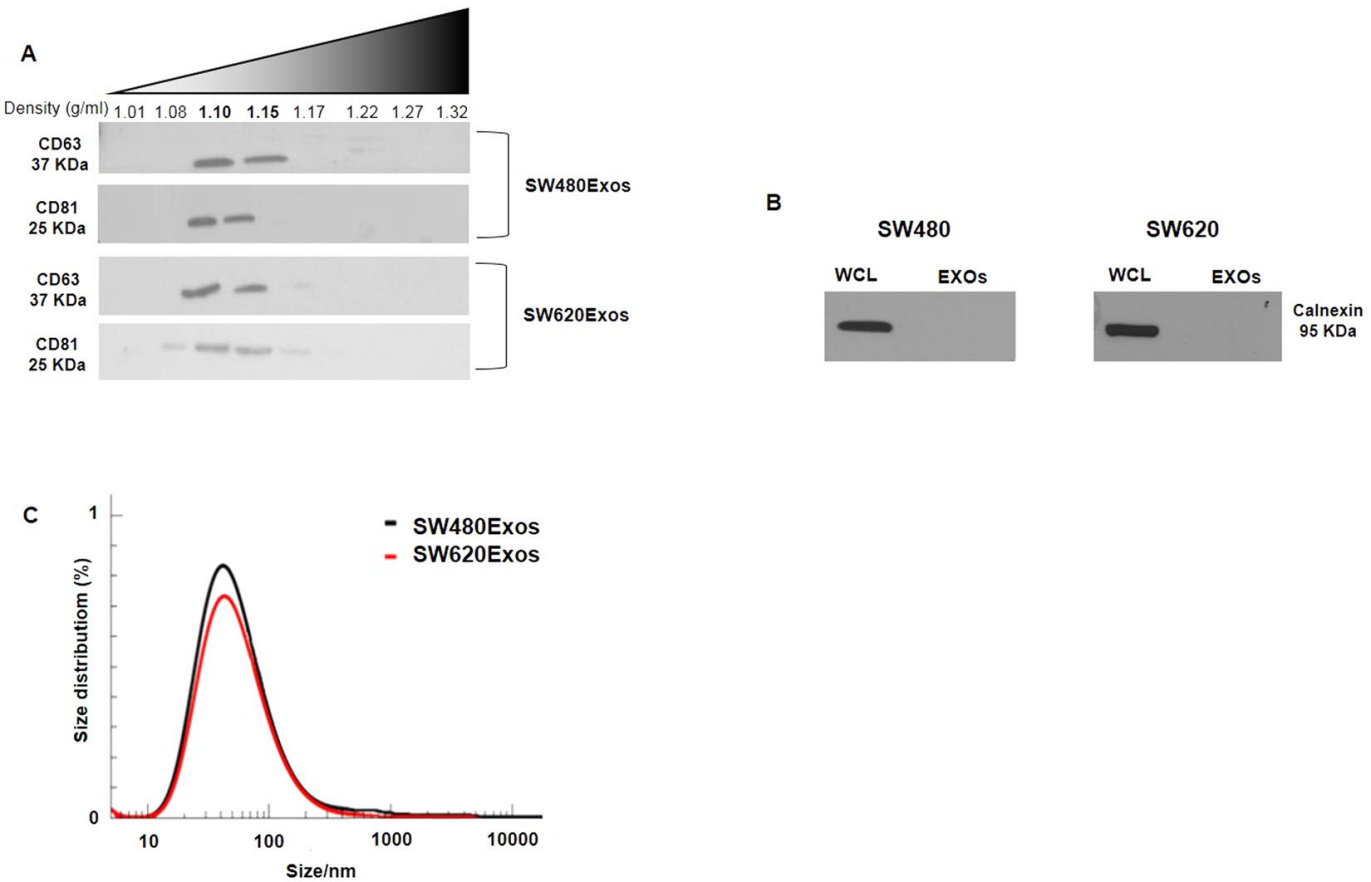
SW480 and SW620 cell-derived exosomes characterization. (**A**) Equal amount (15 µg) of SW480Exo and SW620Exo proteins were probed with the indicated antibodies that detect exosome-enriched proteins. Original uncropped WBs are reported in Figure S7A. (**B**) 30 µg of both SW480Exos and SW620Exos and cellular lysates were incubated with anti-calnexin to exclude cellular contamination in exosome preparation. Original uncropped WBs are reported in Figure S7B. WCL: Whole Cell Lysate; EXOs: exosomes. (**C**) Dynamic light scattering (DLS) analysis of SW480Exos and SW620Exos. Results were plotted as a % mass distribution in order to accurately represent the size distribution of the biological sample.

SW480 and SW620 cell-derived exosomes (SW480Exos and SW620Exos) were purified by flotation in discontinuous 5–60% density centrifugation gradients (Optiprep^TM^) and characterized by dynamic light scattering (DLS) analysis and western blotting (Fig. 1). CD63 and CD81, typically enriched in exosomes^18^, were enriched in 1.10 g/ml and 1.15 g/ml buoyant density fractions, obtained from the gradient fraction derived from the 100,000 × g pellets (Fig. 1A). Moreover, Calnexin, a ubiquitous ER protein, was exclusively found in whole lysate fractions (Fig. 1B). The DLS analysis revealed an average hydrodynamic diameter of about 40 nm for both types of exosomes (Fig. 1C). Collectively, these results show that EVs from SW cells are in the size range of exosomes and express exosome markers.

### Exosomes released by metastatic cells affect morphological and functional properties of non-metastatic tumor cells

**Figure 2:**
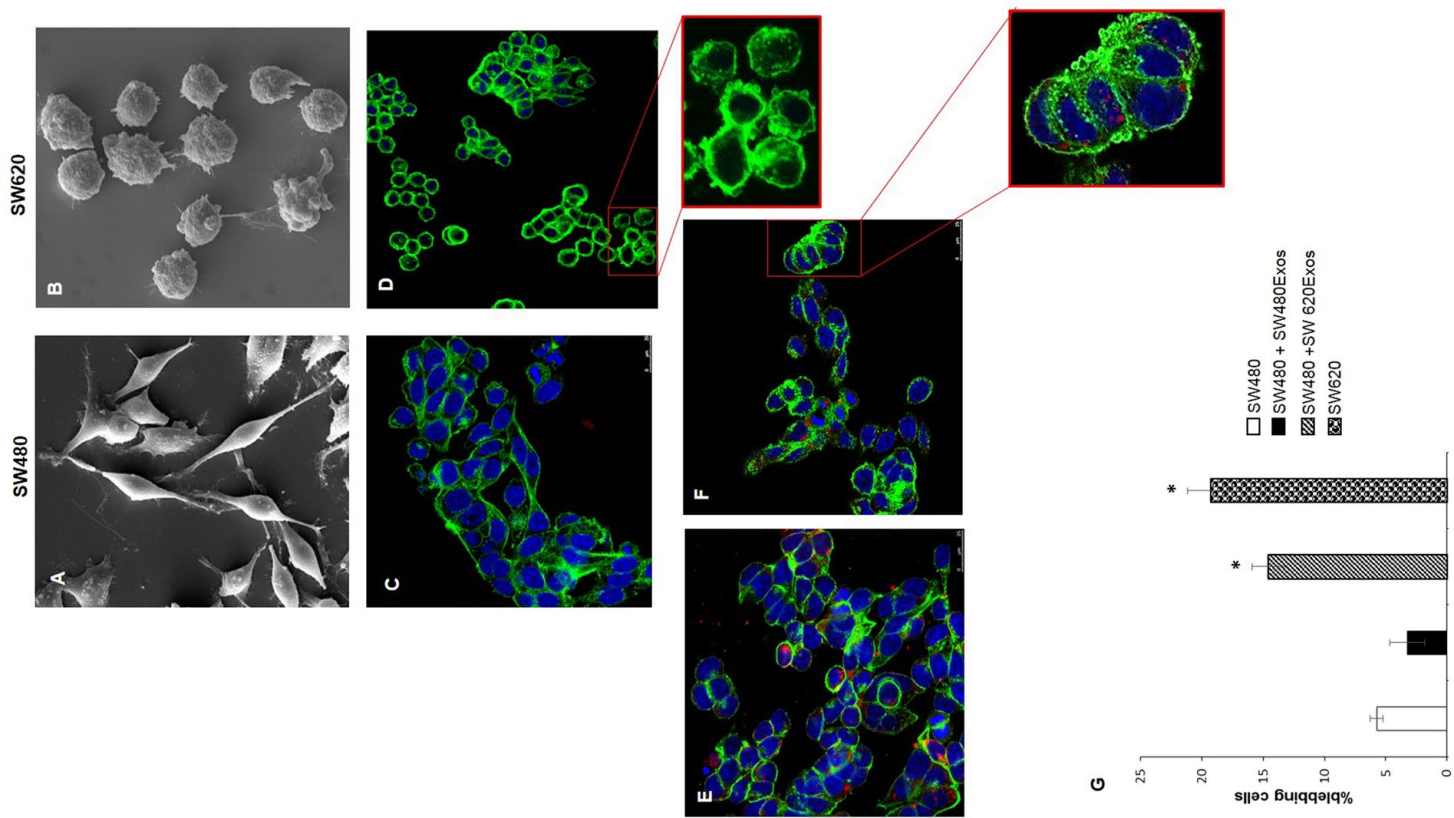
Exosomes released by metastatic cells affect morphological properties of less aggressive tumor cells. Scanning electron microscopy shows the spreading-elongated morphology of SW480 (**A**) and the round-blebbed morphology of SW620 cells (**B**). Confocal micrographs display the different morphology between elongated SW480 (**C**) and amoeboid SW620 cells (**D**) characterized by membrane blebbling (highlighted by magnification). (**E**,**F**) SW480 cells treated for 3 hrs with 20 µg/ml of PKH26-labeled SW480Exos (**E**) and SW620Exos (**F**). Exosomes are visible as red dots inside cells﻿.﻿ Magnification highlights round cells with membrane blebs in SW480 cells after treatment with SW620Exos. Both SW480 and SW620 cells were stained with Actin green (green); nuclear counterstaining was performed using Hoescht (blue). (**G**) Percentage of blebbing cells after exosome treatment and in control condition. The percentage of blebbing cells was obtained by counting 10 different fields for each condition. Values are the mean ± SD of three independent experiments. Significant differences were calculated in comparison to no-treated SW480 cells: *p ≤ 0.05.

**Figure 3:**
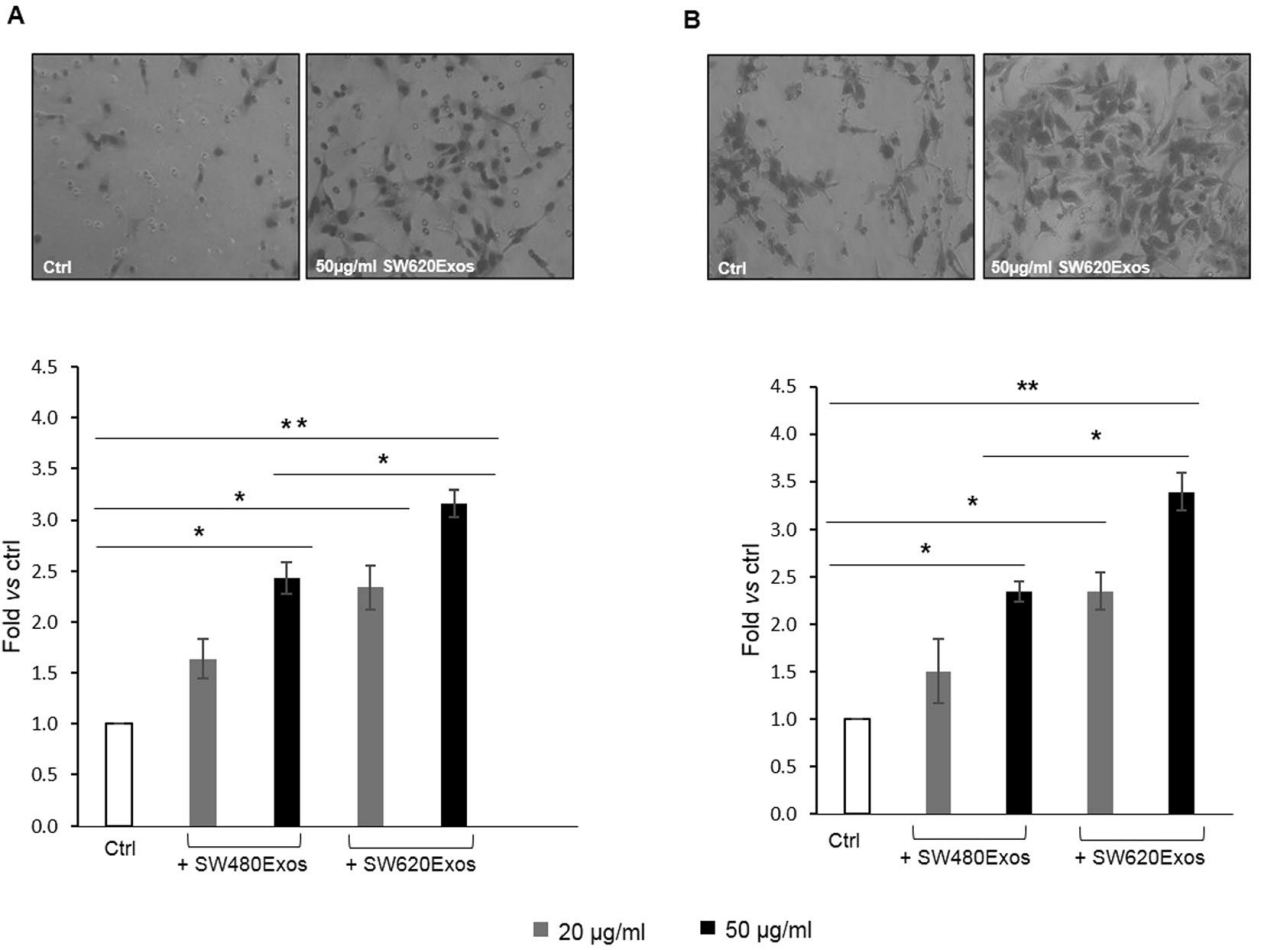
Exosomes released by metastatic cells enhance motile and invasive properties of less aggressive tumor cells. Transwell migration (**A**) and invasion (**B**) assay of SW480 cells treated with increasing doses (20 or 50 µg/ml) of SW480Exos or SW620Exos. Representative micrographs (upper panel) and quantitative data (lower panel) are reported for each assay. Ctrl (Control): SW480 cells without exosome treatment. Values reported in the graphs are the mean ± SD of 5 fields in three independent experiments. Statistical significance was calculated *vs* ctrl and between the same doses of SW480Exos and SW620Exos as indicated by the horizontal lines; *p ≤ 0.05, **p ≤ 0.01.

**Figure 4:**
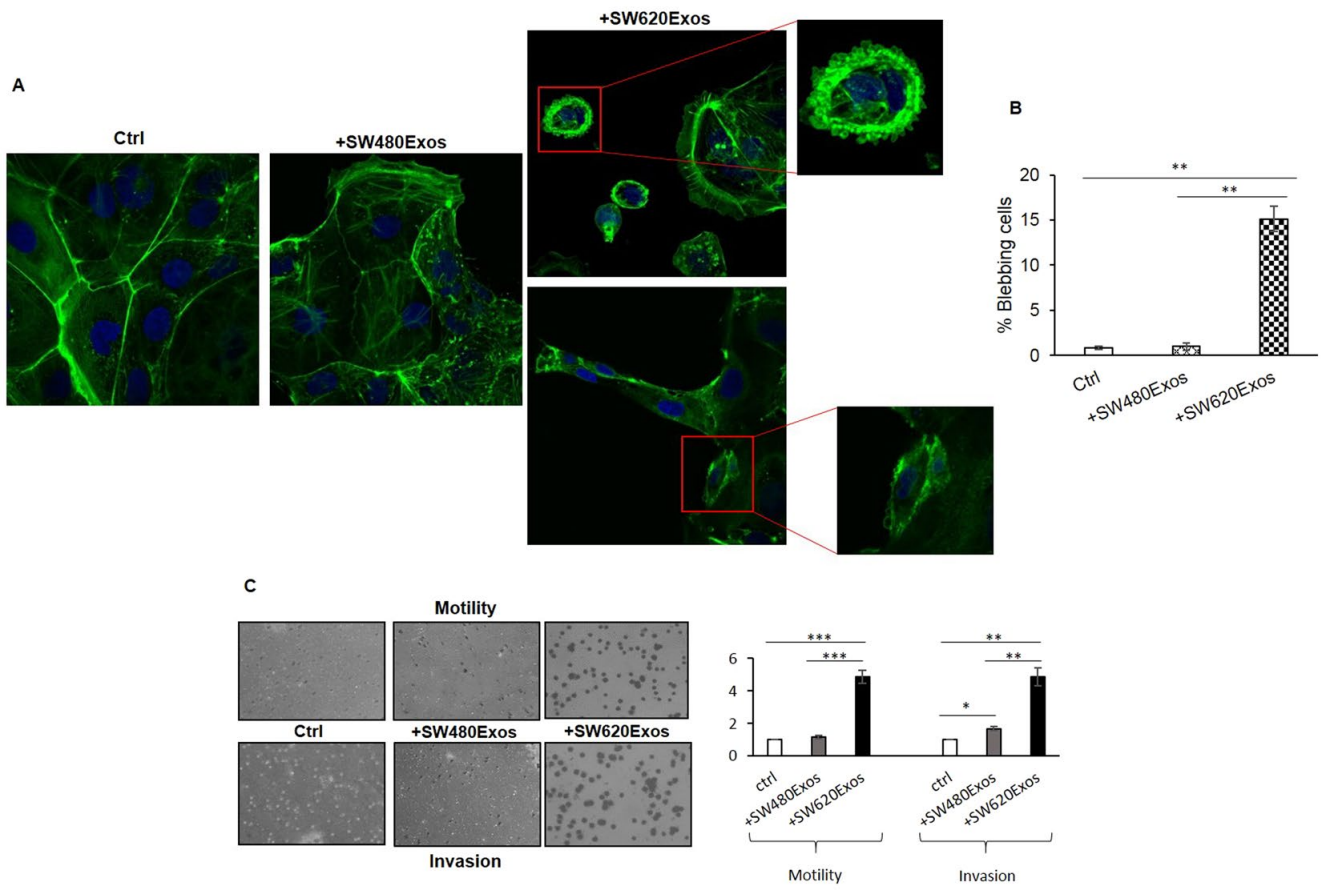
SW620Exos affect morphological and functional properties of Caco2. (**A**) Confocal micrographs of Caco2 cells treated or not for 6 hrs with 20 μg/ml of SW480Exos or SW620Exos. Cells were stained with Actin green (green) and nuclear counterstaining was performed using Hoescht (blue). (**B**) Percentage of Caco2 blebbing cells after exosome treatment and in control condition. The percentage of blebbing cells was obtained by counting 5 different fields for each condition. (**C**) Transwell migration and invasion assay of Caco2 cells treated with 20 µg/ml of SW840Exos or SW620Exos. Representative micrographs (left panels) and quantitative data (right panel) are reported for each assay. Ctrl (Control): Caco2 cells without exosome treatment. Values reported in the graphs are the mean ± SD of 5 fields in three independent experiments. Statistical significance was calculated as indicated by the horizontal lines; *p ≤ 0.05, ** p ≤ 0.01, ***p ≤ 0.001.

SW480 and SW620 cells exhibit different features in culture. In line with published data^19^, our scanning electron microscopy analysis (Fig. 2A,B) indicates that SW480 cells display an elongated morphology (Fig. 2A and confocal analysis in Fig. 2C), whereas SW620 cells appear almost exclusively round (Fig. 2B) with evident membrane blebbing typical of an amoeboid phenotype^20^ (Fig. 2D). By labeling exosomes with PKH26, we observed that SW480 and SW620 cells were able to internalize exosomes after 3 hrs of incubation at 37 °C (Fig. 2E,F and Supplementary Fig. S1). Interestingly, SW620Exos were able to transfer the amoeboid phenotype of the originating cells (Fig. 2D) to SW480 cells (Fig. 2F), inducing a significant increase of the percentage of blebbing cells in this otherwise mesenchymal-like line (Fig. 2G); conversely, the treatment of SW480 cells with their own exosomes did not result in phenotypic changes (Fig. 2E). Furthermore, while SW480Exos did not induce any change in the morphology of SW620 cells, treatment of these cells with homologous exosomes (SW620Exos) induced a propagation of amoeboid features (Supplementary Fig. S1). In order to exclude the possibility that SW620Exos-induced blebbing was due to apoptosis, we performed a caspase 3/7 enzymatic activity assay that demonstrated that SW620Exo treatment does not induce apoptosis in SW480 recipient cells (Supplementary Fig. S2). Finally, because amoeboid membrane blebbing is considered as a morphological feature that is typically associated to rapid motility, which helps cancer cells to quickly migrate in different environments^21^, we tested if the transition of SW480 cells to an amoeboid phenotype coincided with the acquisition of a more aggressive behavior. As shown in Fig. 3 we found that SW620Exos significantly increased, both migration (Fig. 3A) and invasion (Fig. 3B) of non-metastatic cells in a dose-dependent manner. Overall, these results indicate that metastatic cells can transfer their round/amoeboid phenotype to non-amoeboid cancer cells via exosomes and that this morphological transition is associated with the acquisition of a more aggressive behavior. The ability of exosomes released by metastatic cells to transfer a biological and functional phenotype was also confirmed on other non-metastatic colon cancer cells. We found that SW620Exos, but not SW480Exos induced membrane bleb formation and a significant increase of motile and invasive properties also in Caco2 cells (Fig. 4A–C).

### Exosomes derived from cancer cells with different aggressive propensities differentially affect the properties of endothelial cells

**Figure 5:**
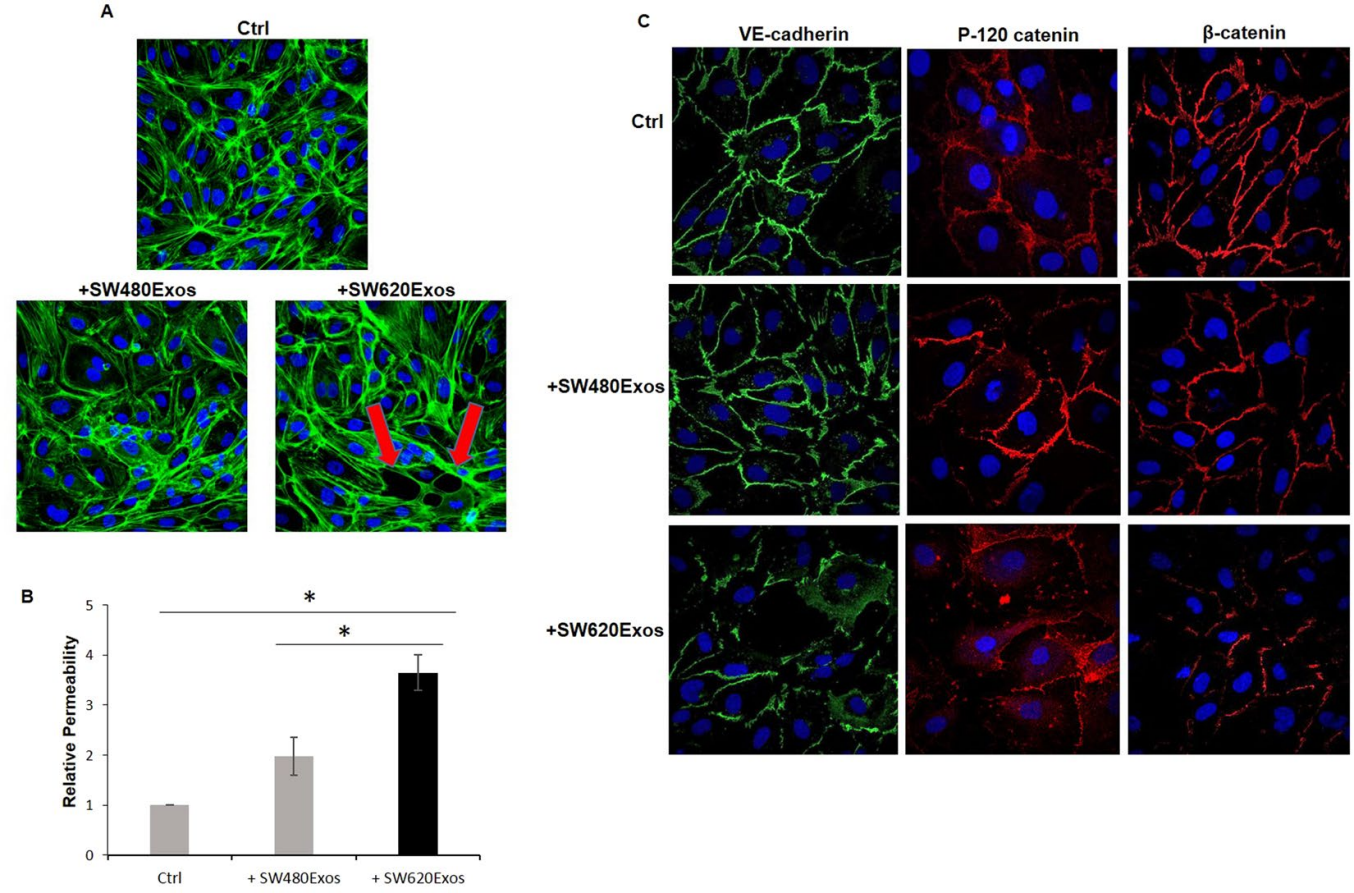
Exosomes derived from cancer cells with different grade of aggressiveness differentially affect the properties of endothelial cells. (**A**) Confocal micrographs of HUVECs treated for 6 hrs with 20 μg/ml of SW480 or SW620 cell-derived exosomes. (**B**) Dextran permeability assay of HUVEC monolayer. Permeability assay was performed by adding in the upper chamber 20 μg/ml of SW480Exos or SW620Exos and 6 hrs after determining FITC-Dextran fluorescence in the lower chamber. Values reported in the graph are the mean ± SD of three independent experiments. Statistical significance was calculated as indicated by the horizontal lines; *p ≤ 0.05. (**C**) Confocal micrographs of HUVECs treated for 6 hrs with 20 μg/ml of SW480Exos or SW620Exos show the localization of VE-cadherin (green), P-120 catenin (red) and β-catenin (red). Nuclear counterstaining was performed using Hoescht (blue). Ctrl (Control): HUVECs treated with vehicle.

Tumor progression is a multistep process due not only to the intrinsic capabilities acquired by tumor cells themselves, but also to their interaction with different components of the tumor microenvironment. In order to understand if exosomes derived from cells with different aggressive propensities can differentially affect cells in the microenvironment, thus contributing to accelerate tumor dissemination, we evaluated the effects of SW480Exos and SW620Exos on the endothelium, by using HUVECs as *in vitro* model. While the ability of HUVECs to internalize SW480Exos and SW620Exos was comparable (Supplementary Fig. S3A), the effects induced by the two populations of exosomes were markedly different. Figure 5A shows that treatment with 20 µg/ml of SW620Exos for 6 hrs affected the integrity of endothelial monolayers resulting in an evident cell leakiness due to the loss of cell-cell contacts, while SW480Exos, used at the same dose and for the same time, caused just a mild alteration of endothelial cell monolayers.

In order to further evaluate the influence of SW620Exos on monolayer integrity we performed an endothelial permeability assay using FITC-dextran molecules. We found that treatment with SW620Exos significantly elevated HUVEC monolayer permeability compared to both the control and the SW480Exo treatment condition (Fig. 5B).

Since it is well known that the regulation of VE-cadherin localization is essential for the endothelial barrier integrity and function^22^, we hypothesized that SW620Exos may regulate the endothelial permeability by changing the expression and/or the localization of VE-cadherin in HUVECs. Our results showed that in comparison to the SW480Exo treatment and the control condition, SW620Exos did not affect the VE-cadherin expression at both mRNA (Supplementary Fig. S3B) and protein level (Supplementary Fig. S3C), but rather induced a clear cytosolic delocalization of the protein, such as of other adherens junction (AJ) proteins, β-catenin and p120-catenin (Fig. 5C). Moreover, we noted that both exosome populations had a comparable positive effect on the ability of HUVECs to form vessel-like structures at least when used at a dose of 10 µg/ml, indicating that both aggressive and less aggressive cancer cells are able to induce angiogenesis through exosome shedding. However, when a higher dose of exosomes (20 µg/ml) was used, we observed that SW480Exos maintained their angiogenic properties, while SW620Exos lost them (Supplementary Fig. S4A,B). These results suggest that the capability of exosomes released by metastatic cells to destroy the endothelium integrity overcomes their angiogenic activity if they are used above a given dose.

Collectively, the above independent assays we carried out for evaluating the effects of exosomes released by spindle versus amoeboid cells demonstrated that exosomes derived from amoeboid cells have a more remarkable ability to increase endothelial permeability. Altogether, these results suggest that inside a heterogeneous tumor mass, different cell sub-populations may differentially support tumor progression through their exosomes, some promoting the formation of new vessels and others inducing endothelial cell leakiness, both indispensable steps of metastatic cascade.

### Comparative proteomic analysis of SW480Exos and SW620Exos

The peptide mixtures obtained after trypsin digestion of three independent SW480Exo (SW480Exo_A, SW480Exo_B, SW480Exo_C) and SW620Exo (SW620Exo_A, SW620Exo_B, SW620Exo_C) preparations were run independently using a Data-Dependent Acquisition (DDA) method. Then, in order to build the spectral library needed for the following SWATH quantitation, all six DDA data sets were integrated. After searching against the Homo sapiens UniProt fasta database by using ProteinPilot 4.5 at a 1% critical false discovery rate (FDR), at both protein and peptide levels, we identified 489 proteins. More than 60% of the proteins were identified based on at least three peptides; proteins were called also with only one peptide but only if this peptide had high confidence (95%) and at least 9 amino acids; the list of identified proteins is shown in Supplementary Tables, *Sheet “Identification MS data”*.

**Figure 6:**
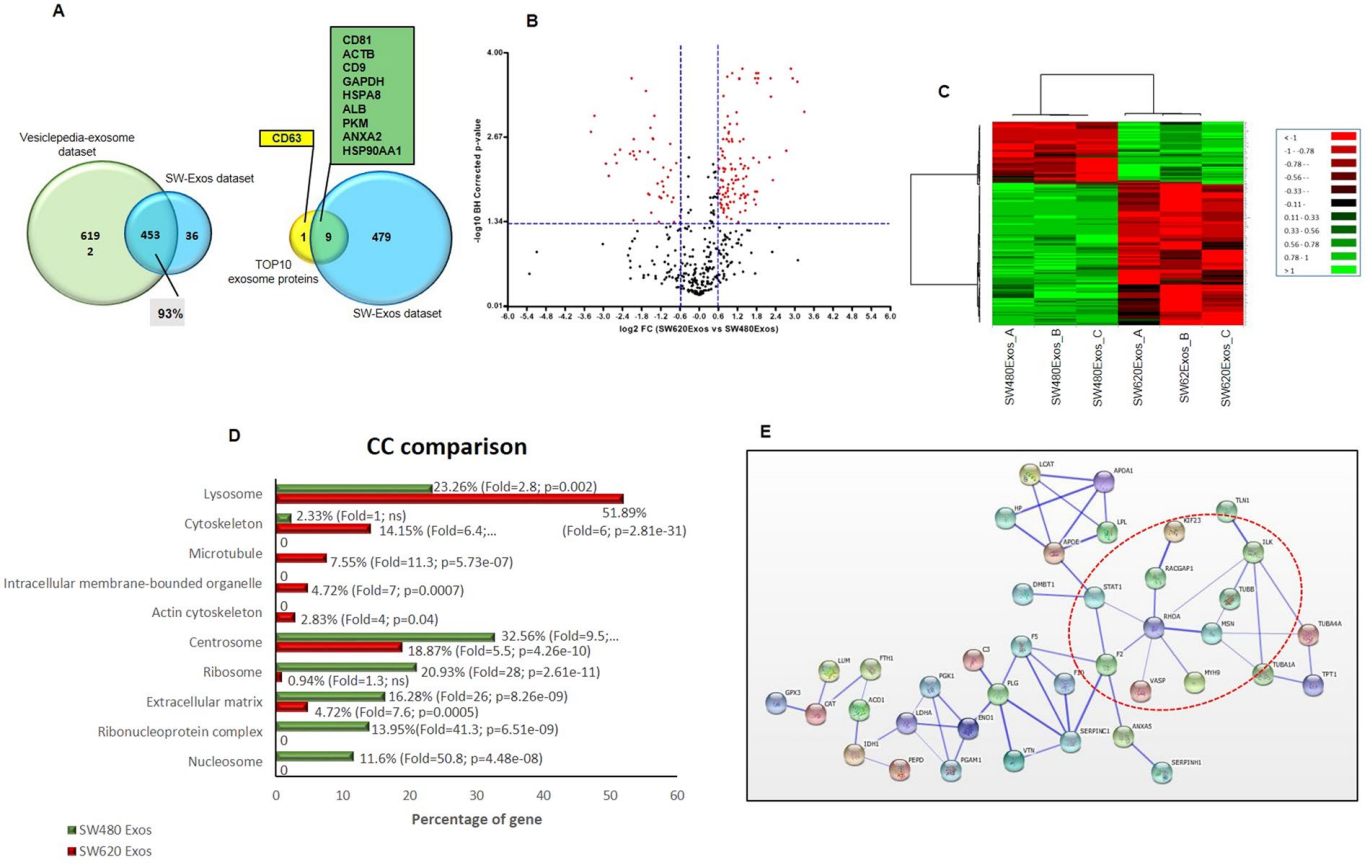
Analysis of protein content of exosomes derived from SW480 and SW620 cells. (**A**) Venn diagrams illustrating comparison of proteins detected in SW-Exos and Vesiclepedia-exosome dataset (on the left) and the Vesiclepedia-Top10 exosome proteins (on the right). (**B**) Volcano plot of the log2 Fold Change SW620Exos/SW480Exos (x-axis) versus the -log10 BH corrected p-value (y-axis) of the 415 quantified proteins. The dashed lines correspond to 1.5-fold up and down (vertical lines), and an FDR value of 0.05 (horizontal line). The red points in the plot represent the 150 proteins that in the two exosome populations are differentially represented with statistical significance. (**C**) Heat map analysis of 150 proteins among three biological replicates between the SW480Exos and SW620Exos. The log10 value of the MS signal intensity is shown. (**D**) Cellular Component (CC) enrichment in SW480Exos and SW620Exos. In correspondence of each bars percentage of gene, fold enrichment and p value are reported; ns: non significant. (**E**) The protein network analysis performed using STRING v10.0 with a confidence level of 0.6 revealed the presence of RhoA partners among the proteins enriched in SW620Exos. The thickness of the connecting line represents the strength of the associations.

In order to assess the quality of our exosome preparations we performed a bioinformatic comparison between our protein dataset and the exosomal protein dataset deposited in the Vesiclepedia database (http://www.microvesicles.org/) by using FunRich (FR), an open access stand-alone tool^23^. We found that more than 90% of the proteins identified in our experiments had been previously described as exosomal proteins (Fig. 6A). Moreover, in our dataset 9 out of the Top 10 exosomal proteins were present (Fig. 6A). Additionally, even if not detected by proteomic analysis, the presence of CD63 was shown in both SW480 and SW620 exosomes (SW-Exos) by western blotting (Fig. 1A). To further characterize the protein content of SW-Exos we also performed a Gene Ontology (GO) enrichment analysis using the FunRich database as a reference. This analysis highlighted that on the Cellular Component (CC) term exosomal proteins were the most significantly overrepresented in our dataset, together with lysosome, centrosome, cytoplasm, mitochondria and nucleus proteins; for the Biological Process (BP), the identified proteins were largely involved in protein metabolism, cell growth and/or maintenance, energy pathway and metabolism while on Molecular Function (MF) analysis, the categories most significantly enriched were extracellular matrix structural constituent, structural constituent of ribosome, protease inhibitor activity, catalytic activity, structural constituent of cytoskeleton and complement activity (Supplementary Fig. S5). Interestingly, these functional classes overlapped with those already described as enriched in other types of TDEs^24^.

In an attempt to highlight differences in protein content between exosomes released by non-metastatic and metastatic cells, we applied the SWATH strategy to quantify proteins content of SW480Exos and SW620Exos. Following ion extraction, peak alignment and normalization were performed using Peakview 2.2 software and the above reference spectral library, resulting in quantitative information for 414 proteins (Supplementary Tables, *Sheet “Quantitative MS data”*).

To identify differentially expressed proteins, a *t*-test analysis was applied, and fold-changes and *p*-values were used to rank and filter the quantitative data. Proteins showing a fold change (FC) ≥ 1.5 in relative abundance and a corrected BH *p*-value < 0.05 were considered differentially represented in the two populations of exosomes (Fig. 6B). In total, we found 150 proteins that were significantly differentially represented between the SW480Exos and SW620Exos and among them 107 were enriched and 43 were depleted in SW620Exos in comparison to SW480Exos (Supplementary Tables, *Sheet “Quantitative MS data”*). The heatmap in Fig. 6C shows the intensity changes of the differentially represented proteins in each biological repeat. All of the differentially represented proteins were functionally categorized using the FunRich software in order to identify the functional classes that were significantly enriched in exosomes derived from metastatic cells but not from non-metastatic cells. As Cellular Component (Fig. 6D), we found that 3 of the five categories enriched in SW620Exos were related to cytoskeletal system: Cytoskeleton (6.4 Fold in SW620 Exos *vs* 1 Fold in SW480Exos), microtubule (11.3 Fold in SW620 Exos *vs* 0 Fold in SW480Exos) and actin cytoskeleton (4 Fold in SW620 Exos *vs* 0 Fold in SW480Exos). Conversely, the majority of the proteins under-represented in metastatic cells-derived exosomes were assigned to the categories of nucleosome (0 Fold in SW620Exos *vs* 50.8 Fold in SW480Exos), ribonucleoprotein complex (0 Fold in SW620 Exos *vs* 41.3 Fold in SW480Exos), extracellular matrix (7.6 Fold in SW620 Exos *vs* 26 Fold in SW480Exos), ribosome (1.3 Fold in SW620 Exos *vs* 28 Fold in SW480Exos) and centrosome (5.5 Fold in SW620Exos *vs* 9.5 Fold in SW480Exos).

The enrichment of cytoskeletal-associated proteins in metastatic cell-derived exosomes was particularly interesting because it is known that the mesenchymal to amoeboid transition as well as the alteration of endothelial structure are specifically related to changes in cytoskeleton organization mediated by the activation of the small GTPase RhoA^25, 26^. Thus, we next asked if within the group of proteins over-represented in SW620Exos there were RhoA interactors able to induce its activation. STRING analysis performed adding RhoA to the dataset of proteins enriched in SW620Exos showed that this GTPase interacts with seven proteins highly represented in metastatic cells-derived exosomes (Fig. 6E). Interestingly among them, RacGAP1 (FC SW620Exos vs SW480Exos = 9.8, p = 6.65e-05; Supplementary Tables, *Sheet “Quantitative MS data”*) and F2 (Thrombin; FC SW620Exos vs SW480Exos = 2.25, p = 7.1e-05; Supplementary Tables, *Sheet “Quantitative MS data”*) are described as direct activators of RhoA^27–30^. In line with the observation that Thrombin was up-represented in SW620Exos, we found that the expression levels of Thrombin mRNA was significantly higher in SW620 cells than in SW480 cells (Supplementary Fig. S6).

### Thrombin exported in SW620Exos is a novel functional mediator of intercellular communication

**Figure 7:**
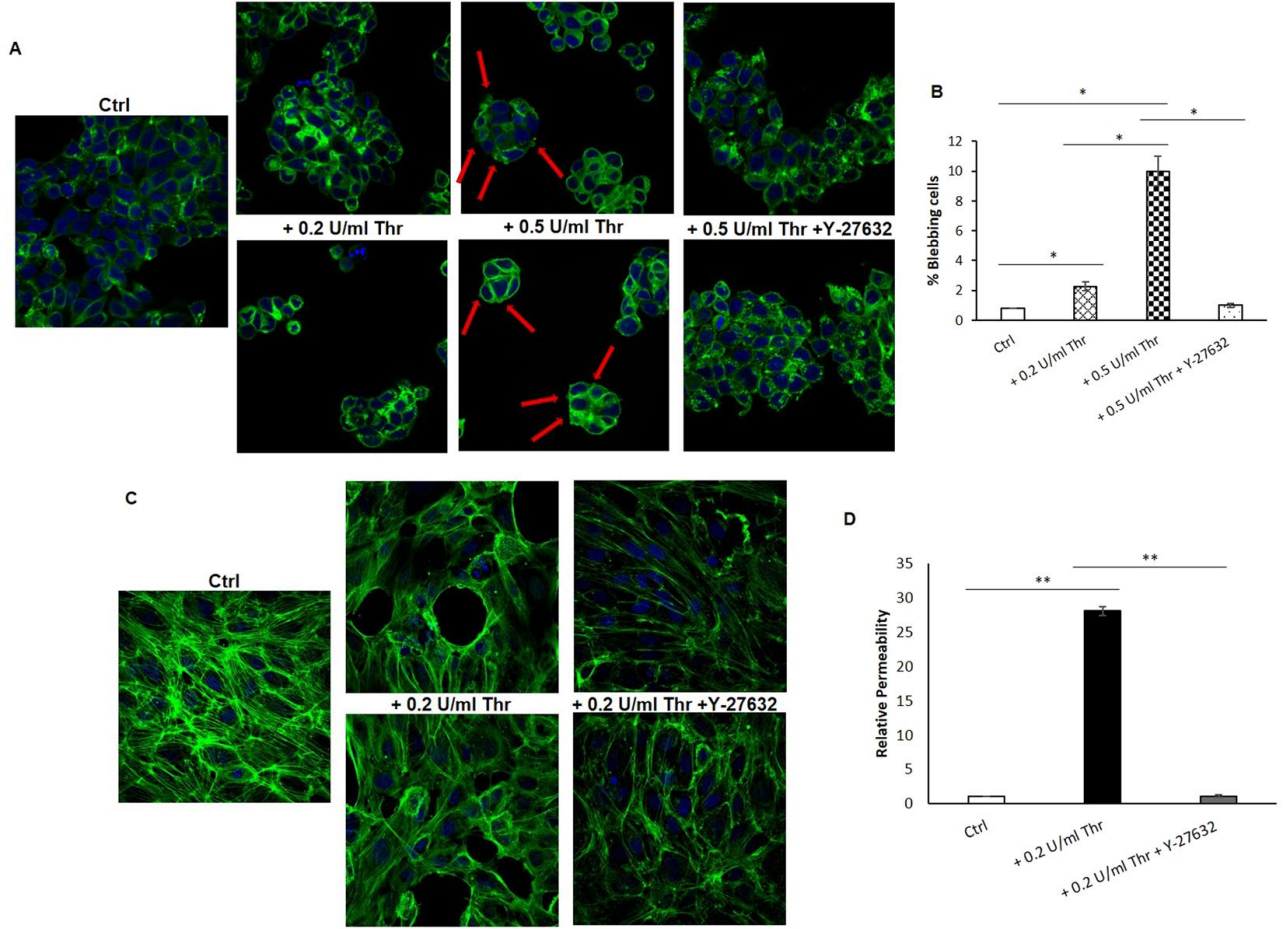
Thrombin is a key mediator of effects induced by SW620Exos in target cells. (**A**) Confocal micrographs show the ability of Thrombin to induce in SW480 cells the transition from an elongated to an amoeboid phenotype in a dose-dependent manner. Moreover, we found that the addition of Y-27632 was able to revert the effect induced by Thrombin, indicating the involvement of RhoA pathway. Except that for the Control (Ctrl: no treated cells), two representative fields are reported for each analysed condition. Cells were stained with Actin green (green) and nuclear counterstaining was performed using Hoescht (blue). (**B**) Percentage of SW480 blebbing cells obtained by counting 5 different fields for each reported condition. (**C**) Confocal micrographs show the ability of Thrombin to alter the HUVEC monolayer. As for SW480 cells, we found that the addition of Y-27632 was able to revert the effect induced by Thrombin. Except that for the Control (Ctrl: no treated cells), two representative fields are reported for each analysed condition. Cells were stained with Actin green (green) and nuclear counterstaining was performed using Hoescht (blue). (**D**) Dextran permeability assay of HUVEC monolayer after treatment with Thrombin and Thrombin + Y-27632. Values reported in the graphs are the mean ± SD of three independent experiments. Statistical significance was calculated as indicated by the horizontal lines; *p ≤ 0.05, **p ≤ 0.01; Thr: Thrombin.

In order to validate the proteomic data and to highlight a key mediator of the phenotypic switch induced in target cells by SW620Exos, we treated SW480 cells with increasing doses of purified Thrombin. Confocal images reported in Fig. 7A show that Thrombin elicited a dose-dependent phenotypic switch in SW480 cells, inducing a rounded morphology at the lower dose (0.2 U/ml) and a significant increasing of bleb formation at the higher dose (0.5 U/ml) as also reported in the graph in Fig. 7B. The transition of SW480 cells from an elongated to amoeboid phenotype following the treatment with thrombin perfectly mimicked the result obtained after exposure to SW20Exos, supporting the central role that thrombin can have in this system. Moreover, we found that when cells were co-treated with thrombin and Y-27632, a specific ROCK inhibitor, the mesenchymal to amoeboid transition induced by thrombin was reverted, strongly suggesting that this effect was mediated by activation of the RhoA/ROCK pathway.

Similarly, we observed that Thrombin induced a remarkable monolayer alteration and a significant increase of endothelial permeability, comparable to that elicited by SW620Exos, in HUVECs (Fig. 7C,D). These effects were reverted when HUVECs were co-treated with thrombin and Y-27632 (Fig 7C,D), again supporting the involvement of the RhoA/ROCK pathway in this process. Our observations support previous reports on thrombin-induced modulation of endothelium permeability mediated by RhoA activation^31^.

### SW620Exos activate the RhoA/Rock pathway in recipient cells

**Figure 8:**
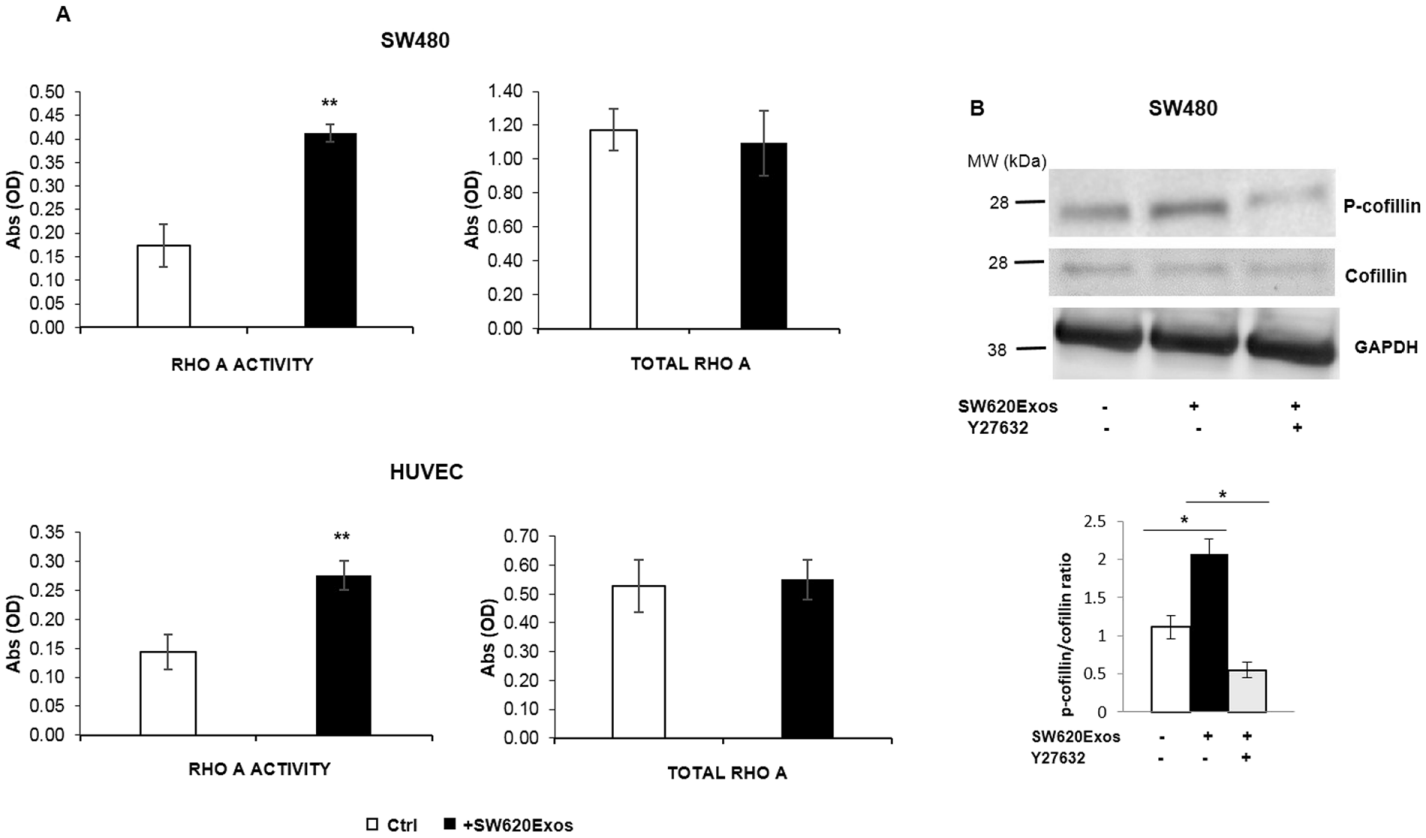
SW620Exos activate the RhoA/Rock pathway in recipient cells. (**A**) RhoA activity (on the left) and total RhoA (on the right) in SW480 cells and HUVECs treated for 6 hrs with 20 μg/ml of SW620Exos. Ctrl: cells without exosome treatment. Values are the mean ± SD of three independent experiments. Statistical significance was calculated *vs* ctrl: **p ≤ 0.01. (**B**) Upper panel reports representative immunoblots showing that treatment with SW620Exos (20 μg/ml) induced in SW480 cells the increase of p-cofilin levels, without affecting total cofilin levels. This effect was reverted by co-treating SW480 cells with the ROCK inhibitor Y-27632 (10 mM). GAPDH was used as loading control. Original uncropped WBs are reported in Figure S7C. The graph in the lower panel shows the ratio between p-cofilin/cofilin normalized to GAPDH. Values are the mean ± SD of three independent experiments. Statistical significance was calculated as indicated by the horizontal lines; *p ≤ 0.05.

In light of the data emerging from the proteomic analysis and evaluating the effects induced by Thrombin treatment on SW480 cells, we wanted to determine if exosomes released by metastatic cells affected the activity of RhoA/Rock signaling pathway in recipient cells. By using the G-LISA RhoA Activation Biochem assay kit, we observed that in both SW480 cells and HUVECs, SW620Exos elicited increased RhoA activity compared with vehicle-treated cells, as demonstrated by a higher amount of RhoA coupled to GTP (Fig. 8A). Levels of active RhoA in SW620Exo-treated cells did not correspond to the levels of total RhoA, and this result was reproduced multiple times (Fig. 8A). Moreover, in SW480 cells treated with SW620Exos we probed the phosphorylation status of cofilin, a downstream effector of RhoA/ROCK. This is an important assay as inactivation of cofilin via phosphorylation is a critical step in actin remodeling. As shown in Fig.8B, we found a clear and significant increase in cofilin phosphorylation in response to SW620Exo treatment. Interestingly, we observed that the positive effect of SW620Exos on cofilin phosphorylation was reverted by co-treatment with Y-27632, a specific ROCK inhibitor (Fig. 8B).

### Inhibition of RhoA/Rock signaling in recipient cells reverts the effects induced by SW620Exos

**Figure 9:**
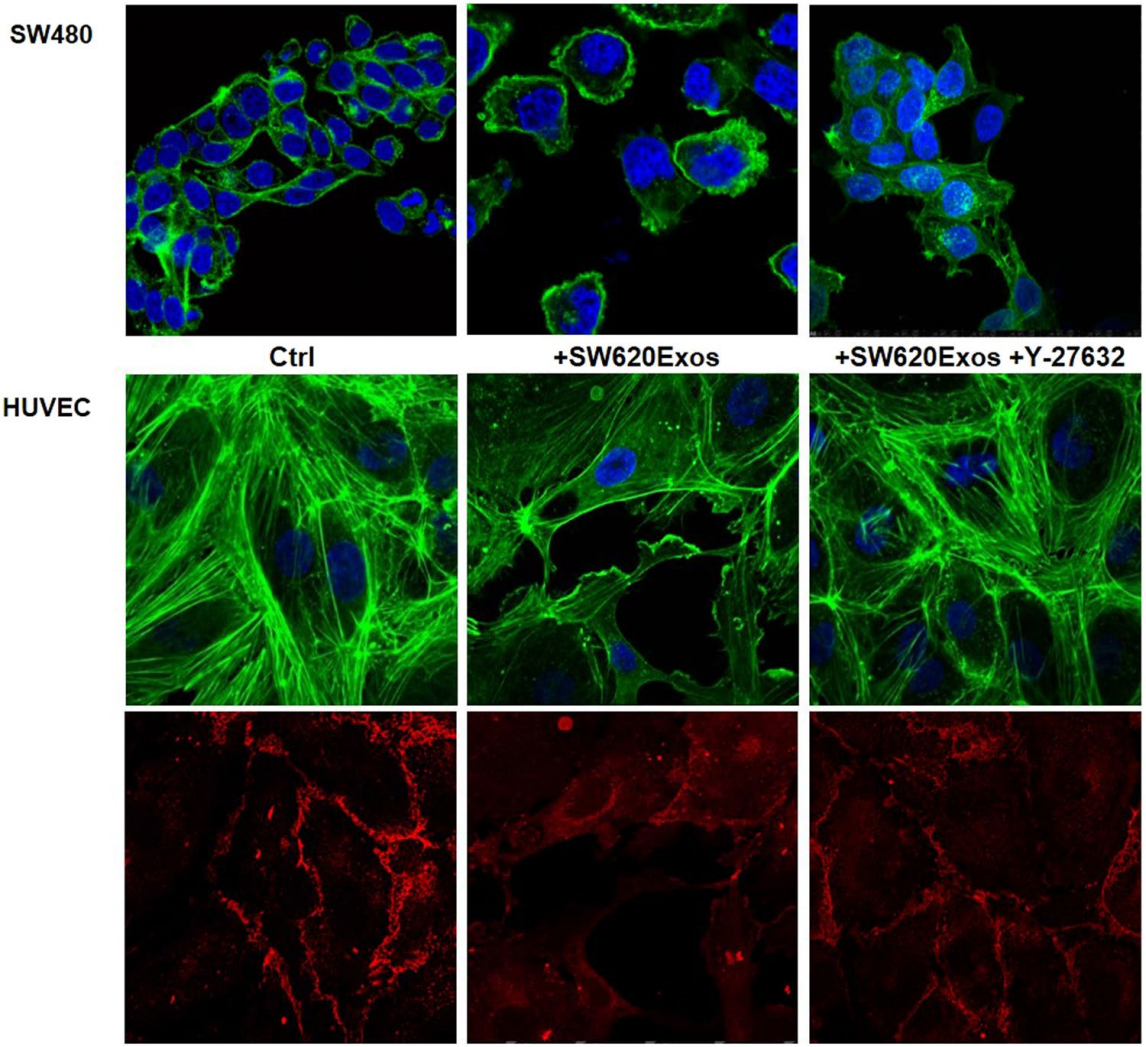
Inhibition of RhoA/Rock signaling in recipient cells reverts the effects induced by SW620Exos. Confocal micrographs show the ability of Y-27632 (10 mM) to revert morphological effects induced by SW620Exos (20 µg/ml) in both SW480 cells and HUVECs. Cells were stained with Actin green (green) and nuclear counterstaining was performed using Hoescht (blue). HUVECs were also probed with anti-VE cadherin (red); Ctrl (Control): no treated cells.

In line with the above result demonstrating the ability of Y-27632 to revert the SW620Exos-induced cofilin phosphorylation status, we found that inhibition of the RhoA signalling pathway by the ROCK inhibitor repressed the SW620Exo-induced morphological and structural effects in recipient cells. The confocal micrographs in Fig. 9 (upper panel) show that, when SW480 cells were co-treated with SW620Exos and Y-27632, the ability of exosomes to induce amoeboid transition and blebbing was completely reverted. Similarly, we observed that the co-treatment with SW620Exos and Y-27632 elicited in HUVECs the recovery of monolayer stability such as the VE-cadherin re-localization to the plasma membrane (Fig. 9, lower panel). Altogether, these results indicate that the effects induced by SW620Exos in both target cells are mediated by the activation of the RhoA/ROCK signaling pathway.

## Discussion

The presence of subclones with distinct genetic, epigenetic and phenotypic properties within a tumor has been widely described and represents one of the major obstacle for a highly efficient and successful prognosis and treatment of cancer^32^. This intratumor heterogeneity is probably due to both genomic instability and dynamicity of tumor microenvironment and is considered one of the main factors that can accelerate the tumor evolution influencing the metastatic potential and therapeutic resistance^33, 34^. Within the complex context of a heterogeneous tumor, sub-clones with distinct characteristics may establish positive interactions resulting in a phenotypic switch that promotes tumor growth and metastasis thus enhancing tumor progression^35^. Several studies clearly demonstrated that the inter-clonal cooperation between metastatic and non-metastatic cells can promote the metastatic cascade at both local and systemic levels^36–38^.

Recent accumulating evidence has demonstrated that within a heterogeneous tumor, tumor-derived exosomes (TDEs) play a relevant role as mediator of the inter-clonal collaborative cooperation^35, 39^. Interesting studies have reported that exosomes released by metastatic cells are able to induce the active transfer in non-metastatic cells of molecules strictly related to the aggressive phenotype (such as EGFRVIII and Met 72 tumor antigen) allowing the recipient cells to acquire abilities typical of metastatic phenotype^12, 40^. Moreover, by using the Cre-LoxP system and intravital imaging it was shown that less aggressive breast tumor cells exhibit increased migratory abilities after uptaking extracellular vesicles originating by highly aggressive cancer cells, indicating that TDEs can act as messengers of malignity^15^.

In the attempt to better define the molecular mechanisms underlying the ability of exosomes released by more aggressive cancer cells to modulate the plasticity of less aggressive sub-clones, we used a unique *in vitro* model of colon rectal cancer (CRC) progression, represented by two isogenic colon cancer cell lines because derived from primary (SW480 cells) and metastatic lesions (SW620 cells) of the same patient^41^. These cell lines represent two different stages of tumor development and can be considered representative of diverse sub-clones that constitute within a tumor the above mentioned condition of heterogeneity. Interestingly these two cell lines show different malignant properties^42^ and are characterized by markedly different morphologies: SW480 cells appear flat and with a spread mesenchymal-like morphology, while the metastatic SW620 cells display an amoeboid phenotype, characterized by rounded shape associated to membrane blebs^20^. Bleb formation has been described as the result of hydrostatic pressure generated in the cytoplasm by the contractile actomyosin^21, 43^, due to a dynamic remodeling of cell cytoskeleton. These morphologies are associated to two distinct modes of motility characteristic of individual tumor cells mentioned as mesenchymal and amoeboid migration. The ability of malignant tumor cells to switch from mesenchymal to amoeboid mode of movement (named mesenchymal to amoeboid transition - MAT) has been widely described^44–46^. This plasticity confers to cancer cells adaptive capacities to microenvironmental changes and is considered a fundamental requirement for tumor progression and metastasis^12^. Moreover, since conversely to the mesenchymal migration the amoeboid motility is independent from MMPs activity and integrin engagement^47–49^, the MAT has important consequences for therapeutic strategies limiting the effectiveness of anticancer treatments based on the use of MMP inhibitors^48, 50^.

In the present study, we obtained evidences that through their exosomes, SW620 cells spread their metastatic abilities to less aggressive isogenic SW480 cells by inducing a phenotypic switch from elongated to rounded amoeboid phenotype associated to higher migratory and invasive capabilities. Notably, while the treatment with SW480Exos resulted, in homologous SW480 cells, in the maintaining of their elongated phenotype, SW620Exos strongly increased the amoeboid phenotype in the metastatic SW620 cells, suggesting the idea that SW620 cells sustain tumor progression both in a paracrine and autocrine manner.

Several recent studies have highlighted that clonal cooperation may facilitate metastasis not only by acting on tumor cell component but also by affecting the microenvironment properties through not mutually exclusive mechanisms^35^. Our data show that exosomes released by SW480 and SW620 cells are able to differentially affect the behavior of the endothelial component. We found that both exosome populations positively affected the ability of HUVECs to form vessel-like structures indicating their angiogenic property. Additionally, the SW620Exos had the exclusive capability to strongly alter the endothelium integrity by inducing an evident loss of the adherens junctions due to the VE-cadherin/catenin internalization, thus overcoming their angiogenic activity. These data suggested that exosomes released by tumor cells with different characteristics within a heterogeneous tumor mass could cooperatively act by promoting formation of new vessels and alteration of their integrity at the same time. These are both indispensable requirements for tumor cells intravasation and dissemination. This cooperative activity may accelerate tumor progression making simultaneous two progressing steps of metastatic cascade.

A pletora of published evidence demonstrates that both MAT and loss of VE-cadherin membrane localization are effects of RhoA activity^46, 51^. RhoA, with Rac1 and Cdc42, belongs to the Rho family of small GTPases that, by cycling between an active, GTP-bound form, and an inactive, GDP-bound form, regulate multiple cellular processes such as growth, survival, and cellular motility driving cytoskeletal reorganization. Several studies have clearly demonstrated that while Rac1 and Cdc42 induce respectively the formation of lamellipodia/membrane ruffles and filopodia^52^, RhoA activation regulates stress fiber formation and actomyosin contraction inducing round morphology and amoeboid motility^46, 47, 53^. For example, it was demonstrated that MDA-MB-231 cells expressing constitutively active Rho G14V mutant exhibited round/amoeboid morphology^54^.

Similarly, it has been reported that VE-cadherin disassembly and vascular permeability can be modulated by mechanical forces and tension exerted on cell adhesion by RhoA signaling inducing actomyosin contractility^26, 55^. Huang and colleagues have demonstrated that sevoflurane prevented LPS-induced rupture of HMVEC-L monolayers by suppressing the VE-cadherin internalization mediated by activation of RhoA/ROCK signaling pathway^51^. Interestingly, Gene Ontology and STRING analyses of SWATH-based quantitative proteomic data obtained by comparing SW480Exos and SW620Exos highlighted that among the proteins specifically enriched in SW620Exos (i) the most represented GO category was that of cytoskeletal associated proteins and (ii) there were seven RhoA interactors. Previous comparative proteomic studies carried out on exosomes derived from SW480 and SW620 cells reported that the major differences between the two nanovesicle populations were related to proteins involved in signaling or with angiogenic properties^56, 57^. The discordance with our results is probably due to the different proteomic strategies used. Our MS data was obtained by applying a SWATH method, a technique in which data-independent acquisition is coupled with peptide spectral library match, and that allows to do label-free quantification in an MRM-like manner. Several recent studies have widely demonstrated that SWATH-MS, combining the strengths of shotgun in terms of proteome coverage, and quantification accuracy and precision of SRM technologies, provides valuable quantification on proteome scale^58, 59^. In light of these considerations, we think that our proteomic results, although limited in terms of proteome coverage in comparison to published data, are robust under a quantitative point of view and more suitable for a comparative proteomic study. As discussed above, in the dataset of proteins over-represented in SW620Exos we found seven RhoA interactors, and among them RacGAP1 and Thrombin (F2) are described in literature as directly responsible for RhoA activation^27, 28^.

RacGAP1 (Rac GTPase-activating protein) is a Rho GTPase-activating protein that plays a key role in controlling various cellular phenomena including cytokinesis, transformation, invasive migration and metastasis^25^. RacGAP1 has the ability to locally suppress Rac1 activity and activate RhoA at the cell front promoting the extension of membrane protrusions and invasive migration, when bound to IQGAP1 (present in both exosome populations; see Supplementary Tables, *Sheet “Identification MS data”*)^25^. The balance between levels of activated Rac and Rho determines the mesenchymal or amoeboid mode of cell motility, and mutual antagonism between Rac and Rho contributes to the maintenance of different modes of cell motility^60^. Therefore, it was notable to find this protein enriched 10-fold in SW620Exos. Moreover, Zhang and colleagues demonstrated that in endothelial cells RacGAP1, by activating RhoA, induced junction breakdown with consequent increase of permeability promoting melanoma cells transendothelial migration^61^.

F2 (Thrombin) is a multifunctional serine protease that converts fibrinogen to fibrin in the blood coagulation cascade and is a potent activator of platelet aggregation. Several studies performed in endothelial and cancer cells have shown that thrombin by PAR signaling induced morphological changes in actin organization through RhoA activation, regulating angiogenesis and cancer progression^28–30, 62^. We demonstrate here, for the first time, that Thrombin, which we found over-represented in SW620Exos, can induce elongated non-aggressive cancer cells to acquire an amoeboid morphology through the involvement of the RhoA/ROCK pathway. In line with these observations, we found that treatment of both cancer and endothelial cells with SW620Exos induced a significant RhoA activation in comparison to the control condition. Moreover, the ability of a specific ROCK inhibitor to revert in both types of recipient cells the effects induced by SW620Exo treatment strongly underlined the central role of RhoA activation in this cellular cross-talk system.

**Figure 10:**
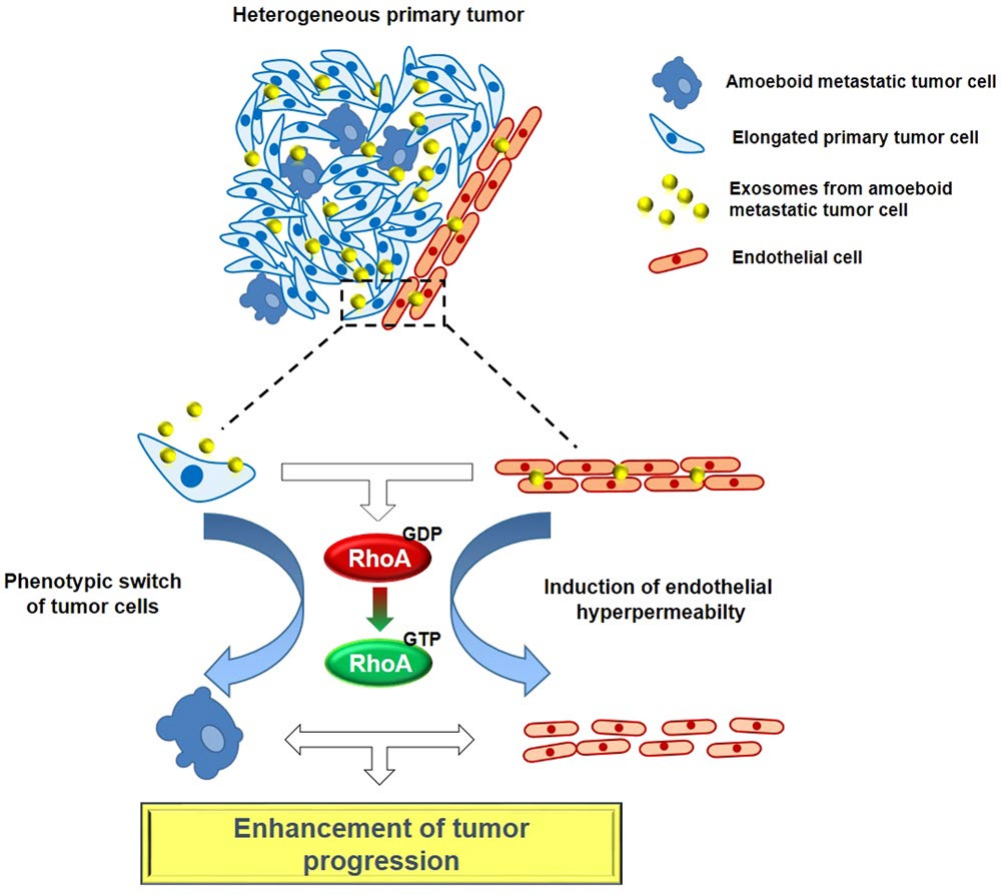
Proposed model of the morphological and molecular effects induced in recipient cells by metastatic cell derived-exosomes.

Our data clearly demonstrate that exosomes can be considered key elements in the context of tumor heterogeneity, because they regulate the collaborative interactions established among sub-clones of cancer cells with different metastatic potentials. In other words, exosomes from more aggressive cells may be able to accelerate tumor progression within a heterogeneous primary tumor, by activating the RhoA/ROCK signaling pathway in cells that uptake them. This process may be able to accelerate tumor progression acting on two distinct levels: 1) by inducing the phenotypic switch of less aggressive tumor cells by transferring the functional properties associated to metastatic behavior; 2) by affecting endothelial stability for the loss of VE-cadherin membrane localization with consequent endothelial hyperpermeabilty (Fig. 10).

In conclusion, our results indicated that, in a heterogeneous context, exosomes released by aggressive sub-clones contribute to accelerate tumor progression by spreading malignant properties that affect both the tumor cell plasticity and endothelial cell behavior. Thus, TDEs might positively regulate metastasis not only by favoring the development of pre-metastatic niches that allow the tumor growth in secondary sites^14^, but also by inducing a rush of the initial steps of the metastatic cascade already in primary tumor site where heterogeneous sub-clones co-exist.

## Materials

### Cell culture and reagents

SW480 and SW620 cells (ATCC), were grown in RPMI 1640 (Euroclone UK) supplemented with 10% fetal bovine serum (FBS; Euroclone UK), 2mM L-glutamine (Euroclone UK), 100 U/ml penicillin and 100 µg/ml streptomycin. Caco2 cells (ATCC), were cultured in DMEM (Gibco USA) supplemented with 10% FBS (FBS; Euroclone UK), 2mM L-glutamine (Euroclone UK), 100 U/ml penicillin and 100 µg/ml streptomycin. Human umbilical vein endothelial cells (HUVEC, Lonza, Clonetics, Verviers, Belgium) were cultured in endothelial growth medium (EGM) according to supplier’s information. SW480 and SW620 cells were maintained at 80% of confluence to recover exosomes. Cells were grown at 37° in a 5% CO2 atmosphere. Where described, SW480 cells were treated for six hours with 0.2 U/ml or 0.5 U/ml of Thrombin. To inhibit ROCK 1/2, cells were treated with 10 µM of Y-27632 ROCK inhibitor (Selleckchem) for 6 hrs. Unless otherwise indicated, all chemicals were from Sigma (St. Louis, MO).

### Scanning electron microscopy (SEM)

SW480 and SW620 cells were seeded and cultured for 48 h as described above. Following removal of medium, cells were fixed with 2.5% glutaraldehyde in 100 mM PBS for 20 min. After dehydration (100% ethanol), the samples were critical point dried (Critical Point Dryer Quorum K8509), gold sputtered (Agar Sputter Coater) and analyzed using a Zeiss, EVO HD15 scanning electron microscope.

### Exosome isolation using OptiPrep™ density gradient medium

Exosomes released by SW480 (SW480 Exos) and SW620 (SW620 Exos) cells during a 24 hrs culture period, were collected from conditioned culture medium supplemented with 10% FBS previously ultracentrifuged (vesicles free media) by different centrifugations, as previously described^63^. Briefly, cells and debris were eliminated by centrifugation at 2,800 g for 10 min; in order to discard large EVs from culture medium, the supernatant was then centrifuged at 10,000 g for 30 min. For isolation of exosomes, the supernatant remaining after the 10,000 g spin was subsequently centrifuged at 100,000 g for 1 hr.

To further purify exosomes, OptiPrep™ density gradient centrifugation of the 100,000 g pellets was carried out^63^. Briefly 60%, 50%, 40%, 30%, 25%, 15%, 10% and 5% solutions were made by diluting a stock solution of OptiPrep™ (60% aqueous iodixanol) in 0.25 M Sucrose/0.9 M NaCl/120 mM HEPES, pH 7.4. The 100,000 g pellet was mixed in the bottom layer and the solutions carefully layered. Centrifugation was performed at 100,000 g for 3 hrs and 50 min at 4 °C with a SW28 Beckman rotor. Eight individual fractions were collected, washed with PBS, and then centrifuged at 100,000 g for 1 h at 4 °C; the pellet from each fraction was then suspended in PBS or lysis buffer. Exosome’s protein content was determined with the Bradford method (Pierce, Rockford, IL, USA).

### Labeling and internalization of exosomes

SW480Exos and SW620Exos were labeled with PKH26, according to the manufacturer’s instructions. Briefly, exosomes were incubated with PKH26 for 10 min at room temperature. washed in PBS, centrifuged, resuspended in low serum medium and incubated with either HUVECs, or SW480 or SW620 cells for 3 hrs at 37 °C. After incubation, cells were processed as previously described^64^ and stained with ActinGreenTM 488 Ready ProbesR Reagent (Life Technologies, USA) that binds F-actin with high affinity. Nuclei were stained with Hoechst (Molecular Probes, Life Technologies, USA). Microscopy was performed using a fluorescence microscope (Nikon Confocal A1). Images were acquired at resolution of 1024 × 1024 and any downstream processing or averaging that enhances the resolution was applied.

### Immunofluorescent microscopy

HUVECs (grown to confluence on coverslips coated with type I collagen - Calbiochem, Darmstadt, Germany), SW480 and SW620 cells (grown to sub-confluence on uncoated coverslip) were treated or not with SW480Exos or SW620Exos (20 μg/ml) for 3 and 6 hrs, Thrombin (0.2 U/ml and 0.5 U/ml) for 6 and 1 hrs and, where reported, co-treated with Y-27632 inhibitor. Caco2 cells (grown to sub-confluence on coverslips coated with type I collagen - Calbiochem, Darmstadt, Germany) were treated or not with SW480Exos and SW620Exo (20 μg/ml) for 6 hrs.

After treatments, cells were processed as described^64^. Antibodies for VE cadherin, β-catenin and p120-catenin (1:100; Santa Cruz Biotechnology, Santa Cruz CA, USA) were used; fuorescently labeled secondary antibodies Alexa Fluor 594 (1:100, Molecular Probes Eugene, Oregon USA) were used.

Actin filaments were stained with ActinGreenTM 488 as above reported. Nuclei were counterstained with Hoechst (Molecular Probes, Life Technologies, USA). The samples were analyzed by using the fluorescence microscope Nikon Confocal A1. Image were acquired at resolution of 1024 × 1024 and any downstream processing or averaging that enhances the resolution was applied.

### RNA extraction and real-time PCR

HUVECs were grown to confluence in 6-well plates and incubated for 6 hrs with SW480Exos or SW620Exos (5–10–20 µg/ml); SW480 and SW620 cells were grown to confluence in 12-well plates for 48 hrs. For quantitative SYBER®Green Real Time PCR, reactions were carried out in a total volume of 20 μl containing 2 × SYBR®Green I Master Mix (Applied Biosystems, Foster City, CA, USA), 2 μl cDNA and 300 nM forward and reverse primers. Primers sequence were: GAPDH (5′ATGGGGAAGGTGAAGGTCG3′, 5′GGGTCATTGATGGCAACAATAT3′), VE-Cadherin (5′GATCAAGTCAAGCGTGAGTCG3′; 5′AGCCTCTCAATGGCGAACAC3′) and Thrombin (5′GCACAGCCAGCATGTGTTCC′, 5′CACATCCGTAGCCGTGGAG3′).

Real-time PCR was performed in duplicate for each data point. Relative changes in gene expression between control and treated samples were determined with the ΔΔCt method. Changes in the target mRNA content relative to GAPDH were determined using the comparative Ct method. Each sample was run in triplicate and Ct means are used for the analysis.

### Western Blot (WB)

HUVEC, SW480, SW620 cell lysates or exosome proteins (15 or 30 μg per lane) were separated using 4–12% Novex Bis-Tris SDS-acrylamide gels (Invitrogen, Life Technologies, USA), transferred on Nitrocellulose membranes (Invitrogen, Life Technologies, USA), and immunoblotted with the following primary antibodies: CD63, CD81, Calnexin, Cofillin, phospho-Cofillin, VE-cadherin (Santa Cruz Biotechnology, Inc., Santa Cruz, CA, USA). All secondary antibodies were obtained from Santa Cruz Biotechnology (Santa Cruz Biotechnology, Inc., Santa Cruz, CA, USA). Chemiluminescence was detected using AmershamTM ECLTM Western Blotting Detection Reagents (GE Healhtcare, UK). The blots were scanned and densitometric analysis performed by Image J software (http://rsbweb.nih.gov/ij/).

### Migration and invasion assay

*In vitro* cell migration and invasion activities were evaluated in transwell chambers as previously described^64^. Briefly, SW480 and SW620 cells (280 × 10^3^/ml) suspended in serum-free RPMI 1640 medium supplemented with 0.1% BSA, with or without increasing amount of exosomes (20–50 µg/ml) were seeded in transwells with 8 μm pore filter coated with collagen (for motility assay) or matrigel (for invasion assay) and exposed to medium supplemented with 10% of FBS (chemoattractant) for 72 hrs at 37 °C in 5% CO_2_. Caco2 cells (20 × 10^3^/ml) suspended in serum-free DMEM medium supplemented with 0.1% BSA, with or without 20 µg/ml of SW480 and SW620 exosomes were seeded in transwells with 8 μm pore filter coated with collagen (for motility assay) or matrigel (for invasion assay) and exposed to medium supplemented with 10% of FBS (chemoattractant) for 48 hrs (for motility assay) and 72 hrs (for invasion assay) at 37 °C in 5% CO_2_. At the end of the assay, after removing non-migrating cells by scraping from the top of the filter, each filter was fixed in ethanol, stained with Diff-Quick (Medion Diagnostics GmbH, Dudingen, Switzerland) and cells were observed by a light microscope at 400x magnification. Each assay was performed in triplicate; migrating/invading cells were counted in at least five high power fields per well by using Image J.

### Dextran permeability assay

HUVECs were seeded onto collagen-coated Transwell inserts (0.4 μm pore size; Corning) at a cell density of 1.5 × 10^5^ cells. After 24 hrs, confluent HUVECs were treated for 6 hrs with 20 μg/ml SW480Exos or SW620Exos, or for 1 h with 0.2 U/ml of Thrombin and then FITC-dextran (2 mg/ml) was added to monolayer (upper chamber) for 1 hrs. FITC-dextran present in the lower chamber was assayed at 495 nm by using GloMax Multimicroplate reader (Promega, Mannheim, Germany). Fluorescence intensity measurements were expressed as Relative Permeability by calculating the fold increase over the basal permeability of untreated monolayer (control).

### Proteomic analyses: sample preparation, SWATH-MS and data analysis

Exosomes were dissolved in 50% TFE/PBS and subjected to tryptic digestion. Three biological replicates of each sample were prepared and subjected to DDA and SWATH analysis. A deep description of the tryptic digestion and DDA/SWATH procedures are reported in Supplementary Material and Methods. DDA raw files were combined and searched against the human database to generate the reference spectral library, which was used for SWATH data processing and quantification. The protein list with FDR lower than 5% generated by analyzing SWATH data with PeakView 2.2, was exported to MarkerView 1.2.1 for statistical data analysis using a pairwise t-test. Three biological replicates were performed for each exosome populations and Fold Change (FC) SW620Exos *vs* SW480Exos thresholds at 1.5 with an adjusted p-value inferior to 0.05 were used to consider a protein up or down-regulated. The p-values were adjusted using a Benjamini-Hochberg (BH) correction and q-value. The FC was transformed using the log2 function, so that the data is centered on zero, while the BH corrected p-value was −log10 transformed for volcano plot scaling. The GO analysis of SW-Exo protein content was performed using the stand-alone enrichment analysis tool FunRich (Functional Enrichment analysis tool; http://www.funrich.org)^23^. The molecular interaction network among the significantly enriched proteins in SW620Exos was analyzed by STRING (Search Tool for the Retrieval of Interacting Genes/Proteins) using the confidence level 0.6. Of note, not all proteins significantly enriched in SW620Exos are included in the presented protein network since disconnected nodes are hidden for better visualizing molecular interaction network.

### RhoA Activation Assay

RhoA activation was quantified by measuring the amounts of RhoA-GTP via the G-LISA RhoA Activation Biochem assay kit (BK150 Cytoskeleton, Inc, Denver, CO), an enzyme-linked immunosorbent assay (ELISA)-based. The total cellular lysates from the SW480 cells and HUVECs treated with 20 μg/ml of SW620Exos or vehicle for 6 hrs were used to measure the RhoA activity. The assay was performed according to the manufacturer’s instructions. Active RhoA-GTP from lysates is bound to the Rho-GTP binding domain of a Rho effector immobilized in the 96-well plate, whereas inactive Rho-GDP is removed during the washing step. RhoA-GTP is subsequently detected by primary anti-RhoA antibody and horseradish peroxidase (HRP)-conjugated secondary antibody. Quantitation of total RhoA was determined by using a RhoA ELISA kit (BK124 Cytoskeleton, Inc, Denver, CO). The HRP reaction signal was quantified by measuring absorbance at 490 nm and recorded using a microplate spectrophotometer (Model 680XR, BioRad). Each sample reading was normalized against the reading of the blank buffer^65^. Each test group was assayed in triplicate.

### Statistics

Data were expressed as means ± SEMs of the indicated number of experiments. Statistical analysis was performed by using a unpaired Student’s t test. Differences were considered to be significant when p-values were ≤than 0.05.

## Electronic supplementary material

Supplementary Materials and Figures

Supplementary Tables of MS data
