# Supplementary material for: Exosomes from metastatic cancer cells transfer amoeboid phenotype to non-metastatic cells and increase endothelial permeability: their emerging role in tumor heterogeneity
Source: Sci Rep. 2017 Jul 5;7:4711. doi: 10.1038/s41598-017-05002-y (PMC5498501; doi:10.1038/s41598-017-05002-y)
Supplement: Supplementary file 1 — Supplementary Materials and Figures [file 41598_2017_5002_MOESM1_ESM.pdf]

Exosomes from metastatic cancer cells transfer the amoeboid phenotype to non-metastatic cells and increase endothelial permeability: their emerging role in tumor heterogeneity

Odessa Schillaci, Simona Fontana, Francesca Monteleone, Simona Taverna, Maria Antonietta Di Bella, Dolores Di Vizio, Riccardo Alessandro

Materials

Dynamic Light Scattering (DLS) analysis

Recovered exosome samples were diluted 30 times to prevent inter-particle interaction and collocated in a thermostated cell compartment at 20°C. Scattered intensity autocorrelation functions g2(t) were measured by using a Brookhaven BI-9000 correlator and analyzed to obtain the distribution P(D) of the diffusion coefficient D. The distribution of hydrodynamic diameter Dh, was estimated by using the Stokes-Einstein relation: $D = (kBT)/(3\pi\eta\ Dh)$, where D is the diffusion coefficient, kB is the Boltzman constant, η is the medium viscosity and T is the temperature. The mean hydrodynamic diameter of exosomes was defined by fitting a Gaussian function to the calculated size distribution.

Tube formation of HUVECs on Matrigel

Matrigel (BD Biosciences) was used to test the effects of exosomes on in vitro vascular tube formation as described1. HUVECs were plated on Matrigel at a concentration of 70000 cells/well in endothelial basal medium containing 0.2% FBS, supplemented with increased amount of exosomes (5-10-20 µg/ml) or EGM as positive control or low serum medium as negative control. Cells were incubated for 4 hrs and then evaluated by phase-contrast microscopy and photographed. The length of the cables was measured manually with the IMAGE-J software.

Proteomics analysis

Protein extraction and digestion. All of the chemicals used for protein extraction and digestion were of analytical grade, and Milli-Q water was employed in all buffers and solutions. After purification about 100 ug of exosomes in 200 µL of 50 % TFE in PBS were vigorously vortexed and sonicated for 2 minutes in an ice bath and finally incubated with constant shaking for 2h at 60°C. Proteins were reduced with 5 mM DTT (Ultrapure-grade, Sigma-Aldrich, Oakville, ON, Canada) for 30’ at 60°C and alkylated with 25mM IAA (Ultrapure-grade, Sigma-Aldrich, Oakville, ON, Canada) for 30’ in the dark at room temperature. Before adding mass spectrometry-grade trypsin (Pierce), the samples were diluted 5-fold with 100 mM ammonium bicarbonate pH 8.0. Protein samples were digested by adding trypsin at a ratio of 1:50 (w/w) for 18 hours at 37°C with constant shaking, in presence of 2mM CaCl2. To stop digestion 20 ul of 90% FA (puriss. P.a. fpr HPLC, Sigma-Aldrich, Oakville, ON, Canada) were added to the samples. Digested samples were then centrifuged at 14,000g for 10 min at 4°C. The resulting supernatant, containing the peptide mixture, was extracted using the 100 µL Bond Elute OMIX C18 pipette tips (Agilent Technologies), according to the manufacturer’s instructions. Eluted peptide mixtures were vacuum dried and reconstituted in 5% acetonitrile 0.1% formic acid for mass spectrometry analyses.

For each sample (SW480Exos and SW620Exos) three biological replicates were prepared for the following proteomic analysis.

Generation of the reference spectral library. Approximately 4µg of tryptic peptides from each of the three biological replicates of the two samples (SW480Exos and SW620Exos) were run for Data-Dependent Acquisition (DDA) analysis. The resulting list of protein/peptides was used for construction of the SWATH reference spectral library. The sample was analyzed via reverse-phase high-pressure liquid chromatography electrospray ionization tandem mass spectrometry (RP-HPLC-ESI-MS/MS) using a TripleTOF® 5600 mass spectrometer (AB SCIEX; Framingham, US). The mass

spectrometer was coupled to a nanoLC Eksigent 425 system (AB SCIEX; Framingham, US). RP-HPLC was performed with a trap and elution configuration using a Nano cHiPLC Trap column 200µm x 0.5mm ChromXP C18-CL 3µm 120Å and a Nano cHiPLC column 75µm x 15cm ChromXP C18-CL 3µm 120Å. The reverse-phase LC solvents were: solvent A (0.1% formic acid in water) and solvent B (2% water and 0.1% formic acid in acetonitrile). The samples were loaded in the trap column at a flow rate of 5 µl/min for 10 min using a solvent, from loading pump, containing 2% acetonitrile and 0.1% v/v TFA in water and eluted at a flow rate of 300 nl/min using a gradient method according to which solvent B is linearly increased from 10% to 28% within 120 min and then to 60% within 30 min; afterwards, phase B is further increased to 95.2% within 2 min. Then, phase B is decreased to 94.8% for 10 min to rinse the column. Finally, B is lowered to 10% over 2 min and the column re-equilibrated for 36 min (200 min total run time). The eluting peptides were on-line sprayed in the Triple TOF 5600 Plus mass spectrometer, that it is controlled by Analysts 1.6.1 software (AB SCIEX, Toronto, Canada).
Each of the six samples used to generate the SWATH-MS spectral library was subjected to a unique DDA run. For these six experiments, the mass range for MS scan was set to m/z 350–1250 and the MS/MS scan mass range was set to m/z 230–1,500. Using the mass spectrometer, a 0.25 s survey scan (MS) was performed, and the top 25 ions were selected for subsequent MS/MS experiments employing an accumulation time of 0.15 s per MS/MS experiment for a total cycle time of 4.0504 s. Precursor ions were selected in high resolution mode (>30,000), tandem mass spectra were recorded in high sensitivity mode (resolution >15,000). The selection criteria for parent ions included an intensity of greater than 50 cps and a charge state ranging from + 2 to + 5. A 15 s dynamic exclusion was used. The ions were fragmented in the collision cell using rolling collision energy, and CES was set to 2.
Six DDA MS raw files were combined and subjected to database searches in unison using ProteinPilot™ 4.5 software (AB SCIEX; Framingham, US) with the Paragon algorithm. The samples were input as unlabeled samples with the following parameters: iodoacetamide cysteine alkylation, digestion by trypsin and no special factors. The searches were conducted through identification efforts in a UniProt Swiss-Prot database (downloaded in July 2014, with 137216 protein sequence entries) containing whole Homo sapiens proteins. A false discovery rate analysis was also performed.
SWATH-MS analysis and targeted data extraction. Six samples (2 µg each) were subjected to the cyclic data independent acquisition (DIA) of mass spectra. Data were acquired by repeatedly cycling through 34 consecutive 25-Da precursor isolation windows (swaths). For these experiments, the mass spectrometer was operated using a 0.05 s survey scan (MS). The subsequent MS/MS experiments were performed across the mass range of 350 to 1250 m/z on all precursors in a cyclic manner using an accumulation time of 0.0898 s per SWATH window for a total cycle time of 3.3335 s. Ions were fragmented for each MS/MS experiment in the collision cell using rolling collision energy, and CES was set to 15. The spectral alignment and targeted data extraction of DIA samples were performed using PeakView v.2.2 (AB SCIEX; Framingham, US) with the reference spectral library. All DIA files were loaded and exported in .txt format in unison using an extraction window of 15 min and the following parameters: ten peptides/protein, seven transitions/peptide, peptide confidence level of 90%, excluded shared and modifies peptides, and XIC width set at 0.05 Da. This export procedure generated three distinct files containing the quantitative output for (1) the peak area under the intensity curve for individual ions, (2) the summed intensity of individual ions for a given peptide, and (3) the summed intensity of peptides for a given protein. For each protein, seven individual ion intensities summed as peptide intensity, ten peptides intensities summed as protein intensity. Mean of all biological replicates was used to compare proteins of the two exosome populations.

References

1. Kohn, E. C., Alessandro, R., Spoonster, J., Wersto, R. P. & Liotta, L. A. Angiogenesis: role of calcium-mediated signal transduction. Proc. Natl. Acad. Sci. U. S. A. 92, 1307–11 (1995).

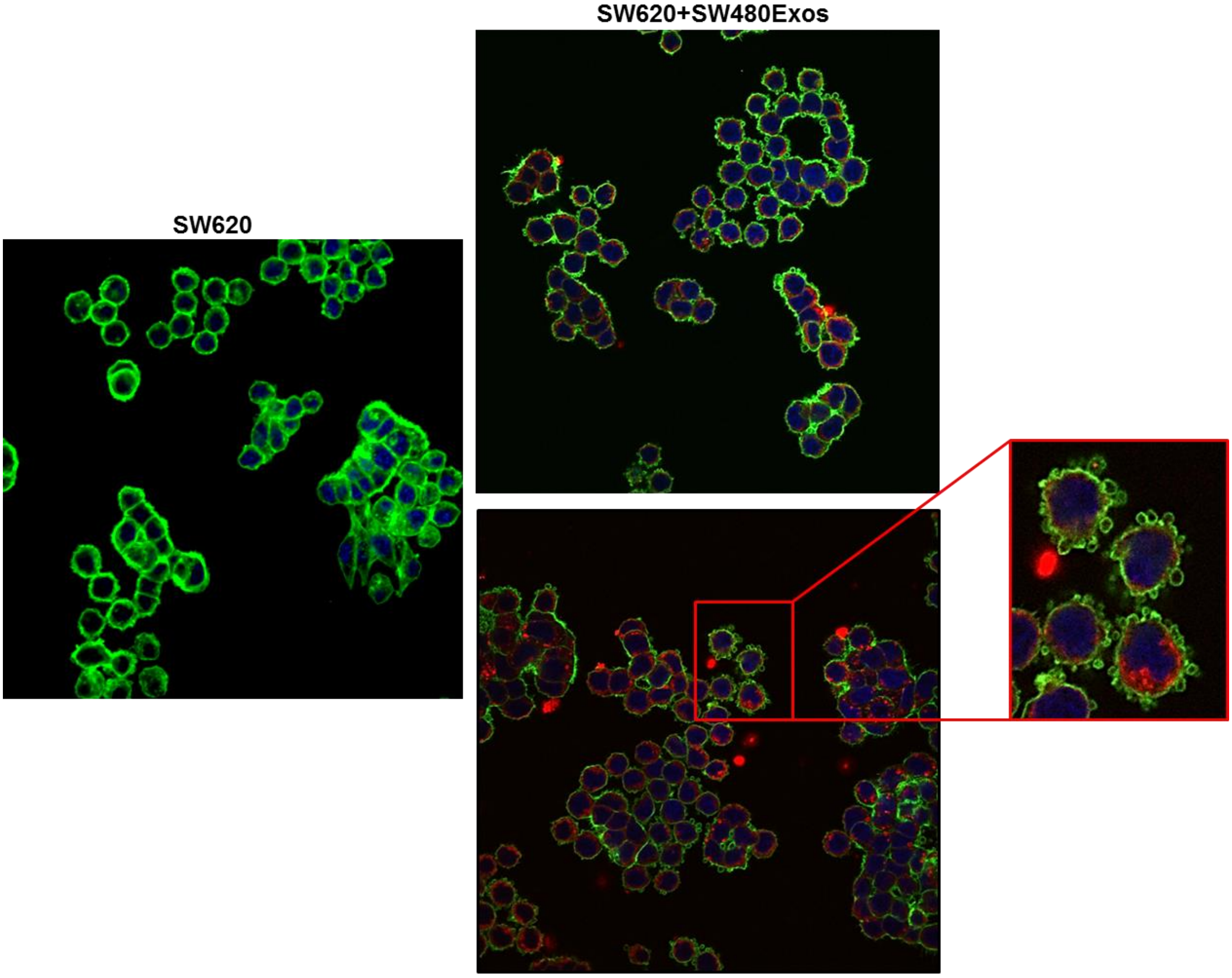

Figure S1. SW480Exos and SW620Exos are uptaken by SW620 cells. Confocal micrographs of SW620 cells treated for 3 hrs with 20 μg/ml of SW480Exos or SW620Exos. Magnification highlights round cells with membrane blebs in SW620 cells after treatment for 3 hrs with their own exosomes. Cells were stained with Actin green (green); nuclear counterstaining was performed using Hoescht (blue) and exosomes were labelled with PKH26 (red dots inside cells). Ctrl (Control): SW620 cells without exosome treatment.

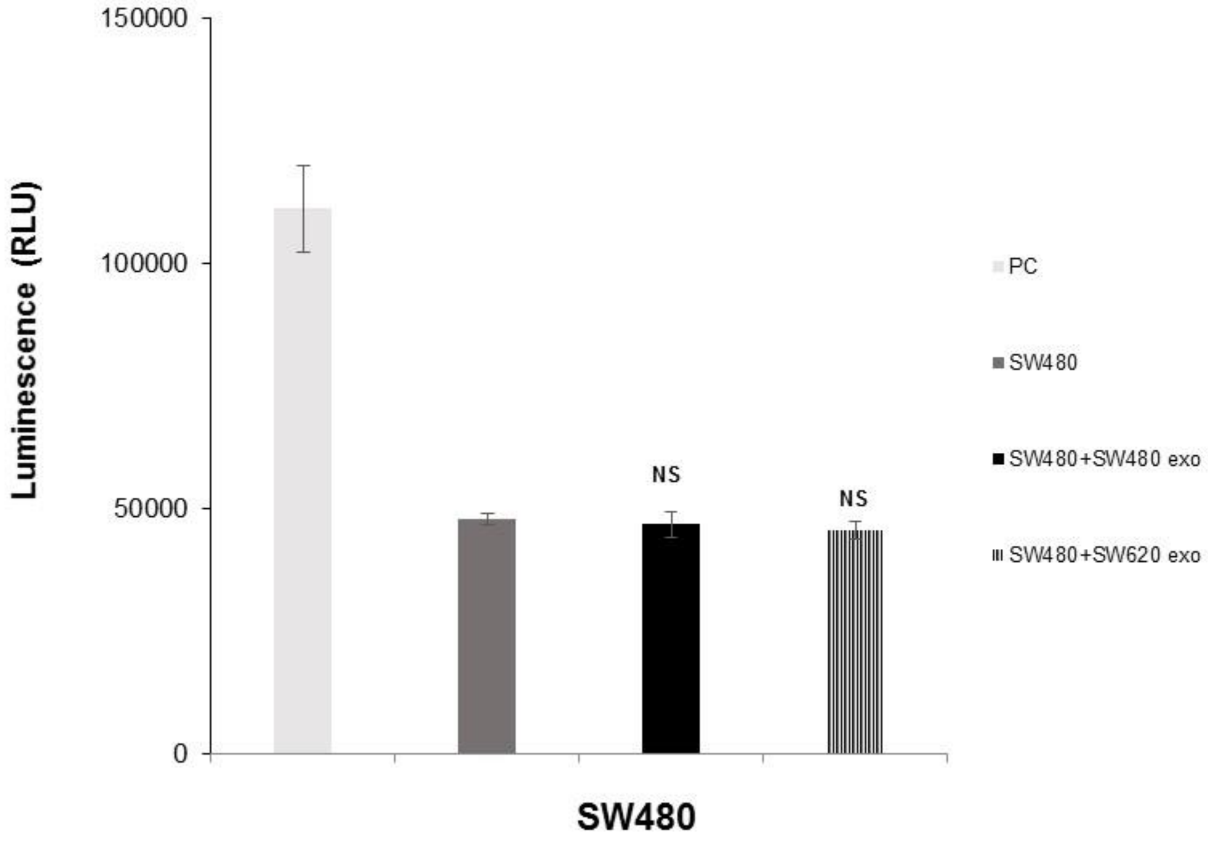

Figure S2. SW620 cell-derived exosomes do not induce apoptosis in SW480 cells. Caspase 3/7 activity assay was performed in SW480 cells treated or not for 6 hrs with 20µg/ml of SW480Exos or SW620Exos. Luminescence was measured using the Caspase 3/7 Glo assay. The reported values are the mean of three independent experiments. PC (positive control of apoptosis): cells treated with 20% DMSO. RLU: Relative Light Unit; NS: no statistical significant differences in comparison to control SW480 cells.

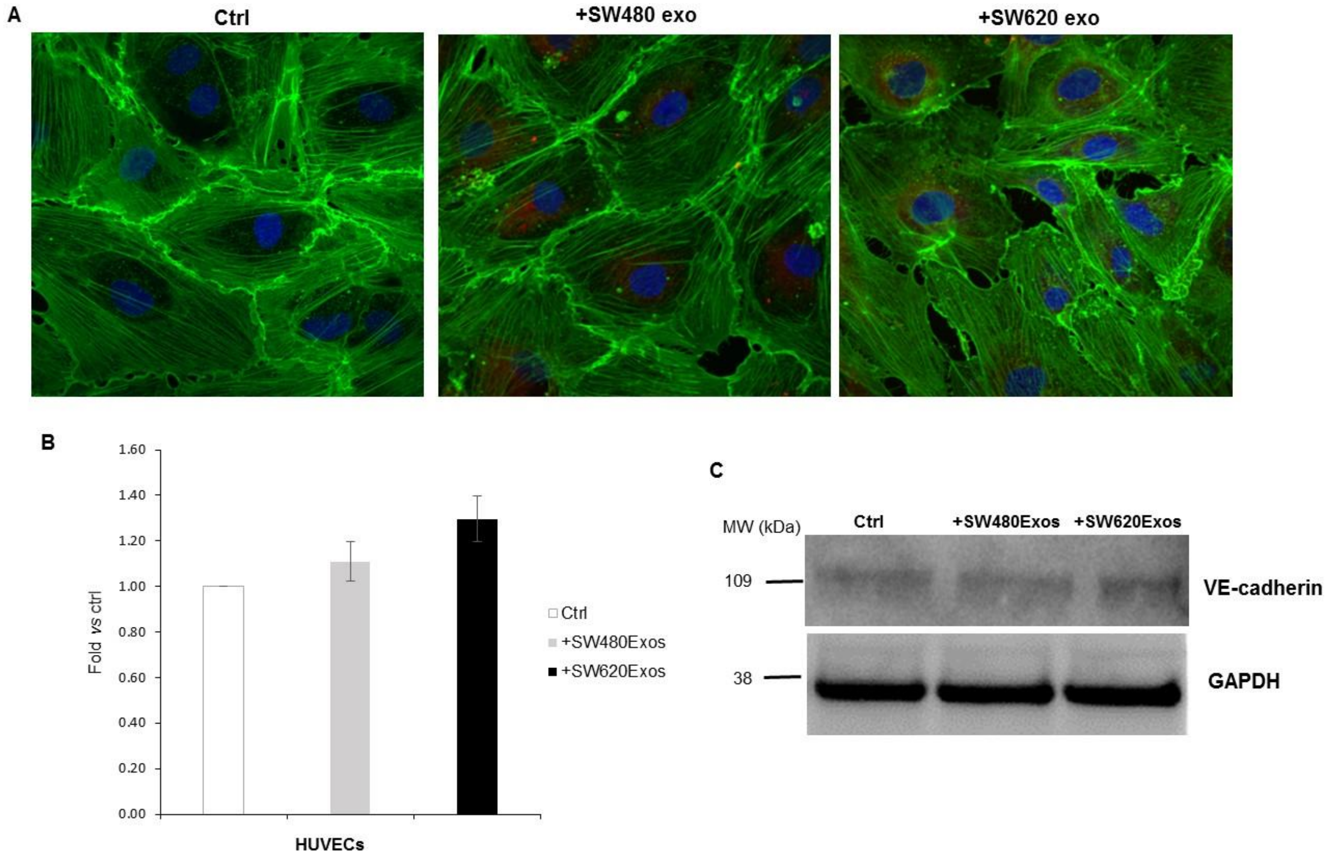

Figure S3. Treatment with SW620Exos does not affect VE-cadherin expression at both mRNA and protein level. (**A**) Confocal micrographs of HUVECs treated or not for 3 hrs with 20µg/ml of SW480Exos or SW620Exos. HUVECs were stained with Actin green (green), nuclear counterstaining was performed using Hoescht (blue) and exosomes were labelled with PKH26 (red dots inside cells). (**B**) Quantitative RT-PCR of VE-cadherin in HUVECs no-treated or treated for 6 hrs with 20µg/ml of SW480Exos or SW620Exos. The reported values are the mean of three independent experiments. (**C**) Representative immunoblot showing that treatment with SW620Exos (20µg/ml for 6 hrs) did not induce variations of VE-cadherin expression. GAPDH was used as loading control. Ctrl (Control): HUVECs without exosome treatment.

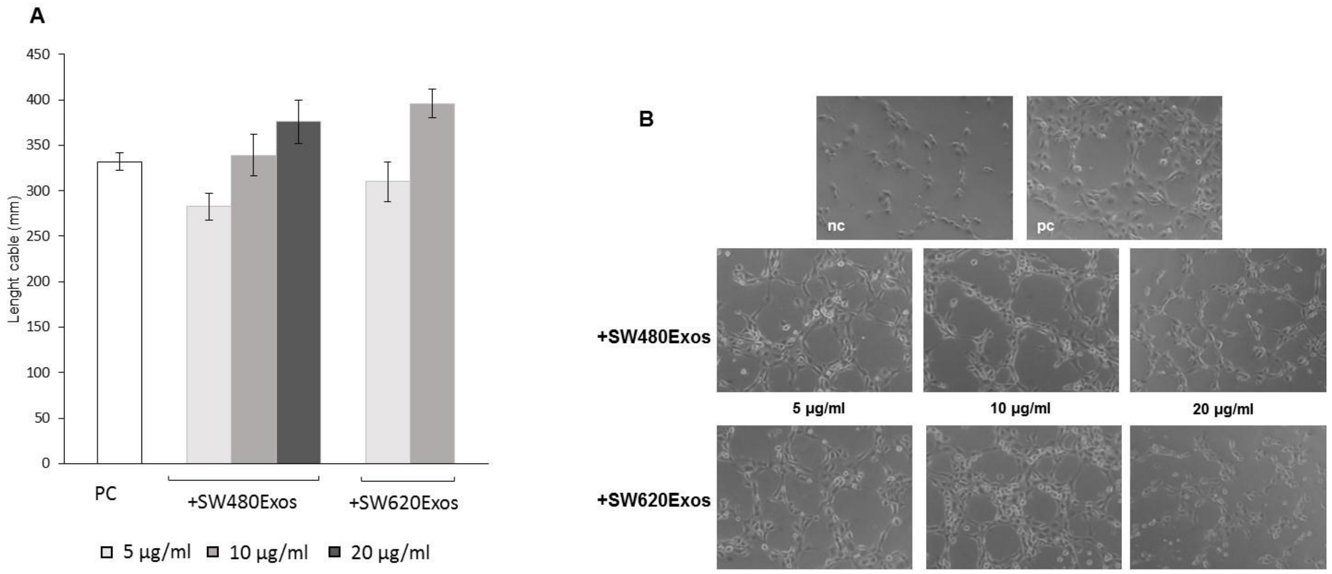

Figure S4. Vascular tube formation on Matrigel highlights the angiogenic properties of both SW480Exos and SW620Exos. (A) Quantitative analysis of the total tube length performed by Image J software. No bar for 20µg/ml SW620Exos is reported because this dose did not induce tube formation, as it is possible to observe in the corresponding micrograph in B. (B) Representative images of HUVECs observed at optical contrast phase microscopy. PC (Positive Control): HUVECs grown in presence of culture medium stimulating tube formation; NC (Negative Control): HUVECs grown in absence of culture medium stimulating tube formation.

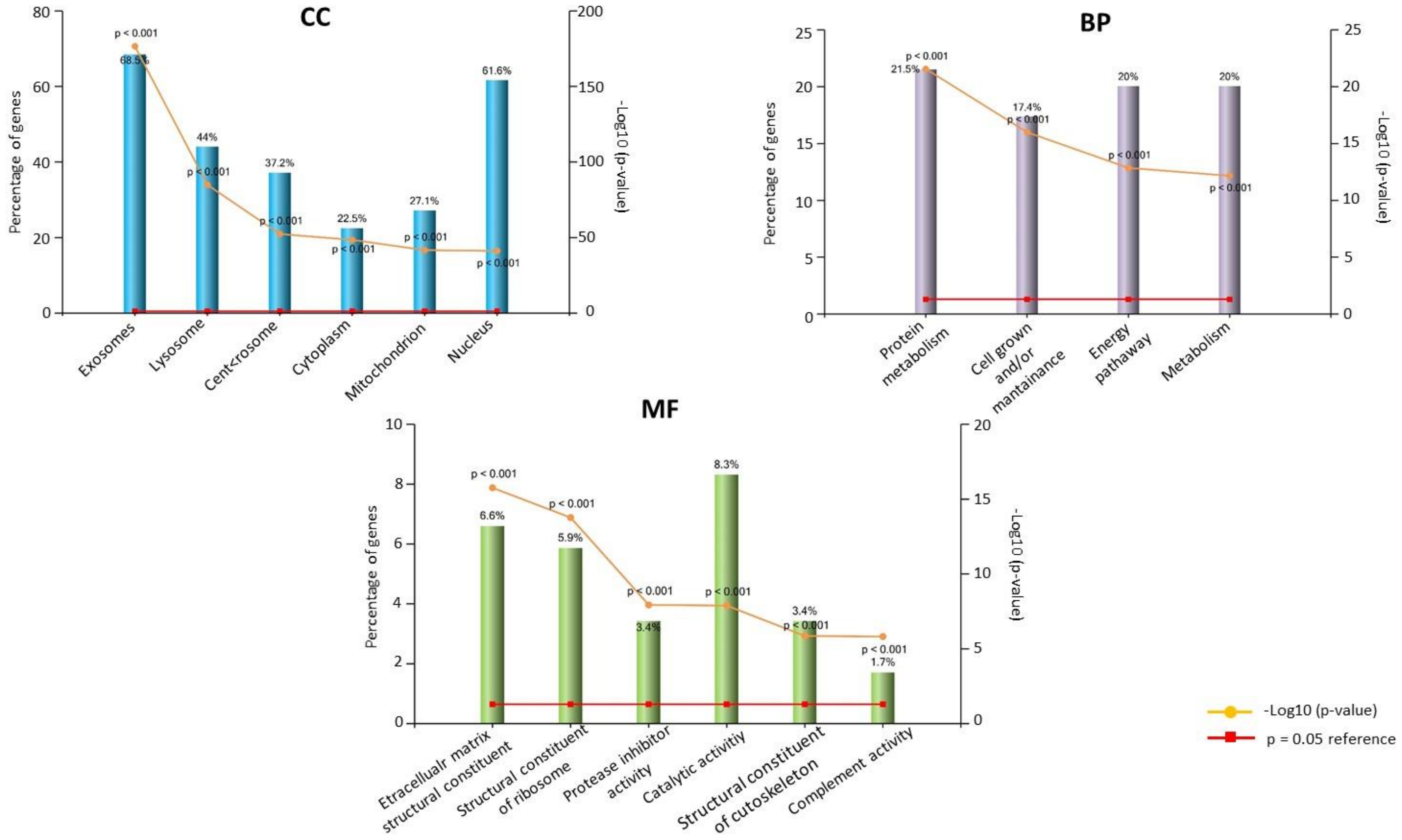

Figure S5. GO enrichment analysis of comprehensive protein content of SW-Exos performed by FunRich. Bar graphs show the most enriched categories in each of the three GO terms: Cellular Component (CC), Biological Processes (BP) and Molecular Function (MF).

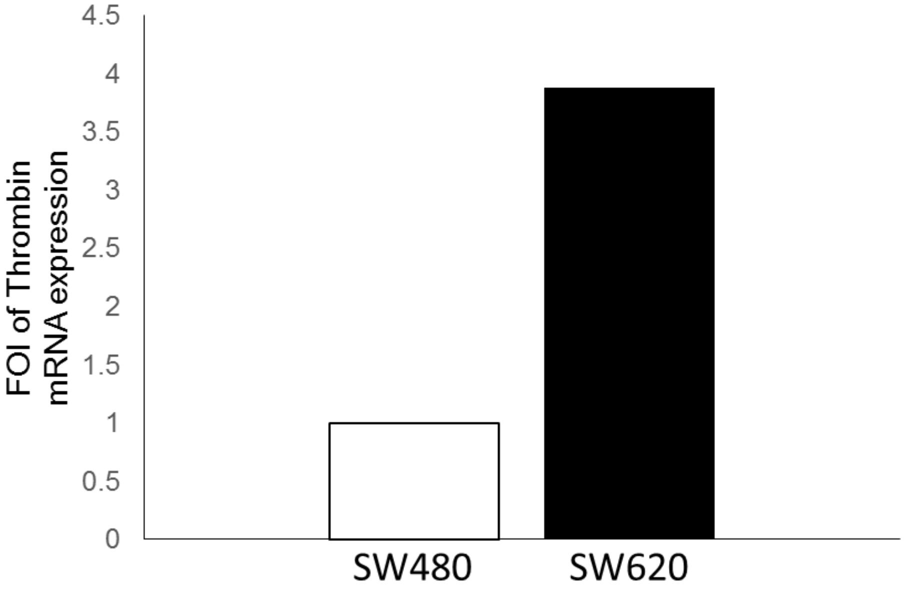

Figure S6. **Quantitative RT-PCR of Thrombin in SW480 and SW620 cells.**

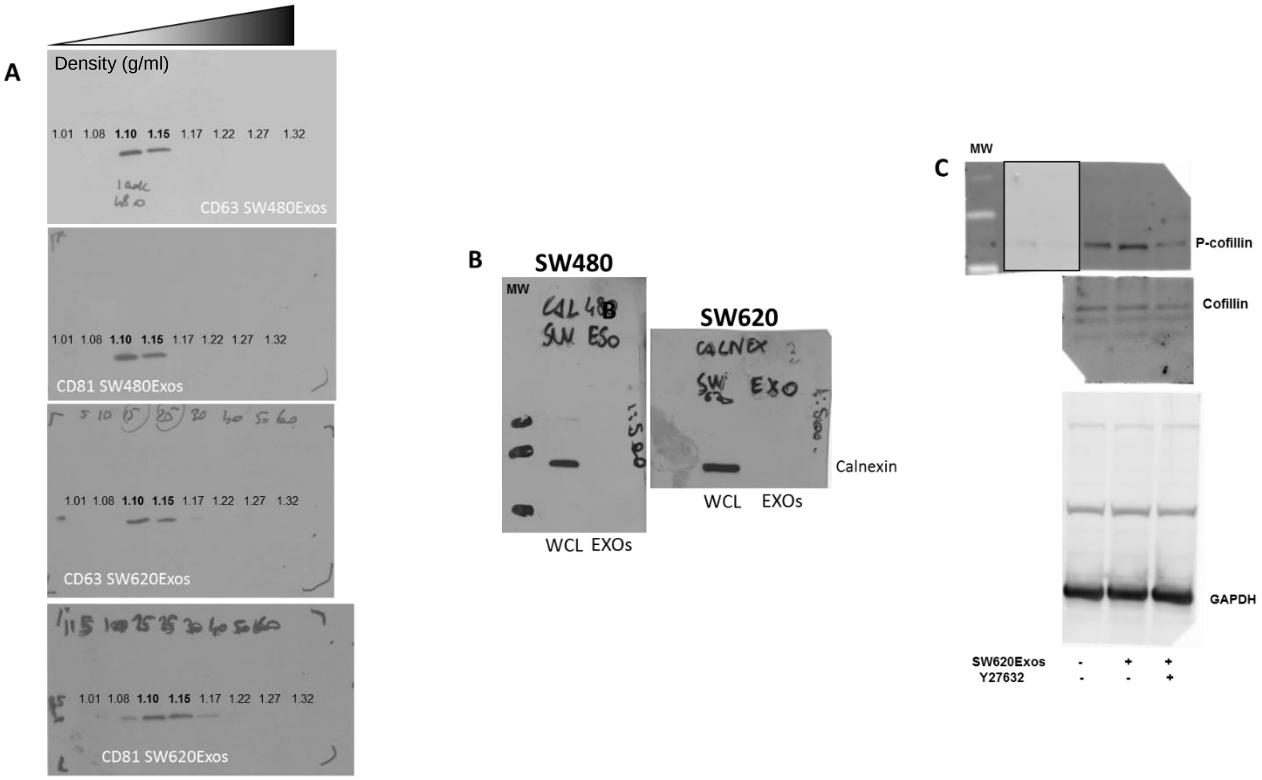

Figure S7. **Original uncropped Western blots** of the images reported in Fig. 1A (A), Fig. 1B (B) and Fig. 8B (C). The white shadow in C covered two samples out of this study.
